# 50 years of rational‐emotive and cognitive‐behavioral therapy: A systematic review and meta‐analysis

**DOI:** 10.1002/jclp.22514

**Published:** 2017-09-12

**Authors:** Daniel David, Carmen Cotet, Silviu Matu, Cristina Mogoase, Simona Stefan

**Affiliations:** ^1^ Babeş‐Bolyai University, Departament of Clinical Psychology and Psychotherapy and International Institute for Advanced Studies of Psychotherapy and Applied Mental Health; ^2^ Icahn School of Medicine at Mount Sinai

**Keywords:** efficacy, irrational beliefs, meta‐analysis, rational beliefs, REBT

## Abstract

**Objective:**

Rational emotive behavior therapy (REBT), introduced by Albert Ellis in the late 1950s, is one of the main pillars of cognitive‐behavioral therapy. Existing reviews on REBT are overdue by 10 years or more. We aimed to summarize the effectiveness and efficacy of REBT since its beginnings and investigate the alleged mechanisms of change.

**Method:**

Systematic search identified 84 articles, out of which 68 provided data for between‐group analyses and 39 for within‐group analyses.

**Results:**

We found a medium effect size of REBT compared to other interventions on outcomes (*d =* 0.58) and on irrational beliefs (*d* = 0.70), at posttest. For the within‐group analyses, we obtained medium effects for both outcomes (*d* = 0.56) and irrational beliefs (*d* = 0.61). Several significant moderators emerged.

**Conclusion:**

REBT is a sound psychological intervention. Directions for future studies are outlined, stemming from the limitations of existing ones.

Rational emotive behavior therapy (REBT) is the original form and one of the main pillars of cognitive‐behavioral therapies (CBT). Alongside with the cognitive therapy (CT) created by Aaron Beck (1976), it served as the basis for the development of CBT. Albert Ellis first introduced REBT in 1957 with the name of rational therapy (RT); later, to emphasize its focus on emotional outcomes, it was named rational emotive therapy (RET). Finally, in the 1990s, Ellis changed its name into rational emotive behavior therapy (Ellis, 1995) because behavioral factors constitute a fundamental component of this treatment approach. More recently, practitioners and scholars started to call it rational‐emotive and cognitive‐behavior therapy (e.g., see the training certificate of the Albert Ellis Institute) to emphasize its role in the CBT paradigm.

In REBT, irrational beliefs are considered central factors of emotional distress, so the focus is on *changing irrational beliefs into rational beliefs*, with the aim of changing dysfunctional emotions and maladaptive behaviors into functional and adaptive ones. The REBT protocols are similar in structure to other CBT approaches (e.g., CT protocols), the main difference relying in the targeted beliefs: REBT specifically focuses on *evaluative beliefs*, (appraisals) and not inferential or descriptive ones (which is often the case with CT; see for details David, Lynn, & Ellis, 2010).

In practice, REBT has been applied in various domains like clinical psychology, education (e.g., rational emotive education), organizational settings (e.g., rational emotive coaching, rational effectiveness training), or counseling (e.g., rational pastoral counseling); (David, 2014). Empirical research in REBT practice has initially focused on effectiveness studies (i.e., how REBT works in real intervention settings) and used mainly transdiagnostic outcomes (i.e., not related to specific psychiatric conditions); thus, few efficacy‐oriented controlled studies (i.e., research in rigorously controlled conditions, using randomized control trials), which are required for a therapeutic approach to be considered evidence‐based, were conducted in the early age of REBT (David, 2014). Since the late 1980s and early 1990s, however, REBT has been investigated in a series of randomized control trials that proved it efficacious for a variety of conditions such as obsessive‐compulsive disorder (Emmelkamp & Beens, 1991), social phobia (Mersch, Emmelkamp, Bogels, & van der Sleen, 1989), depression (David, Szentagotai, Lupu, & Cosman, 2008), side effects of breast cancer treatment (Montgomery et al., 2014; Schnur et al., 2009), psychotic symptoms (Meaden, Keen, Aston, Barton, & Bucci, 2013), parental distress (Joyce, 1995), and disruptive behavior (Gaviţa, David, Bujoreanu, Tiba, & Ionuţiu, 2012).

Beyond the meta‐analyses in which REBT was included in the category of CBT (Cuijpers et al., 2013) or psychotherapy in general (Shapiro & Shapiro, 1982), REBT research data have been summarized so far by four meta‐analyses, all of them indicating REBT as an effective form of psychotherapy (Engels, Garnefsky, & Diekstra, 1993; Gonzalez et al., 2004; Lyons & Woods, 1991; Trip, Vernon, & McMahon, 2007). However, the most recent meta‐analysis on REBT interventions with adults is older than 20 years (Engels et al., 1993), while the most recent ones on REBT interventions with children and adolescents are about 10 years old (Gonzalez et al., 2004; Trip et al., 2007). Moreover, the previous meta‐analyses suffer from certain shortcomings. First, they included a relatively small number of controlled studies (e.g., the Gonzalez et al.’s meta‐analysis included only 19 studies, while Engels et al.’s included 28 studies), which limits their conclusions. Second, they were focused on outcome analyses alone, thus providing efficacy and effectiveness data, but no explicit inquiry on the alleged mechanisms of change (i.e., rational and irrational beliefs).

Knowing whether REBT particularly affects rational and irrational beliefs is important, because validating psychological treatments implies, along with the treatment package validation, the validation of the underlying theory (David & Montgomery, 2011). In this sense, knowing why an intervention works is equally important to knowing if it is effective. Therefore, an updated, sound summarizing of the existing data on REBT efficacy is equally needed and timely.

The current meta‐analysis aims to provide a comprehensive, up‐to‐date quantitative review of REBT, examining whether there is robust empirical evidence for (a) the efficacy / effectivness of REBT interventions, (b) its effect on the alleged mechanisms of change (i.e., rational and irrational beliefs), and (c) the relation between alleged mechanisms of change and intervention outcomes. We considered rational and irrational beliefs *alleged mechanisms of change* because, according to REBT theory, the change in *irrational and rational beliefs* (i.e., what we conceptualized as mechanisms of change) leads to changes in dysfunctional emotions and maladaptive behaviors (outcomes), so that makes them *theoretical* mechanisms of change. This idea has been advanced and tested before in the literature, in both experimental research and clinical trials (Szentagotai, David, Lupu, & Cosman, 2008). However, in this meta‐analysis, we did not directly test the mediation hypothesis of irrational beliefs because this would have required data based on mediational designs, which have been rarely used in clinical studies; rather, we looked at the association between effect sizes in the proposed mechanisms and targeted outcomes. We also set to analyze other relevant moderators of the effect sizes.

## 1 METHOD

### 1.1 Literature search

We located the potential relevant studies by an extensive literature search conducted through the following databases: PsychINFO, PubMed, Scopus, and Web of Science up to January 2015. We used the following key search terms: “rational emotive,” “rational‐emotive,” “rational therapy,” “rational intervention,” “rational counseling,” “rational education,” “rational effectiveness training,” “RET,” “EREC,” “REBT,” “Albert Ellis,” “rational beliefs,” “irrational beliefs.” In addition, we checked theoretical reviews of the REBT literature and preexisting meta‐analyses (Engels et al., 1993; Gonzales et al., 2004; Trip et al., 2007) and identified several additional studies.

### 1.2 Selection of studies

The search strategy produced a total number of 2164 potentially relevant articles. After removing duplicates and irrelevant entries, a total of 586 full‐text articles were assessed for eligibility. We used the following inclusion criteria: (a) studies included an intervention derived from the REBT devised by Albert Ellis (irrespective of the way it was labeled in the study), (b) sufficient data were provided to allow effect size computation, and (c) studies were published in English in peer‐reviewed journals. Some authors (e.g., Emmelkamp & Beens, 1991) labeled the specific intervention they tested as “cognitive therapy” or “CBT,” albeit the intervention protocol included irrational beliefs restructuring based on the REBT model; when this was the case, we included the study in the meta‐analysis. We excluded 502 studies for not complying with the aforementioned criteria.

A total number of 82 studies from 84 distinct articles remained to be included in the meta‐analysis. The differences in counts were given by one article that included two different studies (Thorpe, Freedman, & McGalliard, 1984) and five articles that reported data on two different samples (David et al., 2008; Sava, Yates, Lupu, Szentagotai, & David, 2009; Szentagotai et al., 2008 for one sample, and Thurman, 1985a,b for the second sample). Out of these studies, 68 studies provided postintervention or follow‐up data for the comparative (between groups) analysis, and 39 provided within‐group data for pre‐post or pre‐follow‐up analysis.

For each of the included studies, we retained the following information: identification data (author, year of publication); comparison condition (type of control; see coding details below); outcome, assessment time point (pre/postintervention, follow‐up); effect size data; and a series of moderator variables. We grouped the specific *outcome measures* in the following categories: behavioral outcomes; cognitive outcomes (other cognitions than rational and irrational beliefs); emotional outcomes (comprising anger, anxiety, depression, and distress); health outcomes; psychophysiological outcomes; quality of life; school performance; social skills; and others, which could not be classified into the above categories and were too few cases to form categories by themselves (e.g., study skills measures, parenting measures). Emotional outcomes are also presented separately, in a more specific level of analysis.

The alleged mechanisms of change were grouped into rational beliefs and irrational beliefs (conceptualized as such by the authors). We analyzed rational and irrational beliefs together because the REBT intervention targets both processes–decreasing irrational beliefs and strengthening rational beliefs; for meta‐analytic purposes, we coded them according to the intervention scope (i.e., smaller values for irrational beliefs after the intervention and, respectively, higher values for rational beliefs were all coded as positive effect sizes).

Based on the existing literature (for details, see David et al., 2010), as *moderators*, we coded the following: (two evaluators had to agree 100% on the coding)

- Type of REBT intervention (psychotherapy, educational, counseling): We considered the intervention as being psychotherapy if the authors specifically used and detailed an REBT protocol used with clinical or subclinical population; being an educational intervention if the main pillars of REBT were taught in a class setting; and being a counseling intervention if the intervention, regardless of setting, was delivered to nonclinical population and targeted nonclinical outcomes (e.g., increasing performance but not decreasing depression).
- Type of data report: self‐report, clinician‐report, reports from sources other than the clinician (e.g., a parent), and objective report (included physiological data and behavioral performance objectively quantified).
- Type of control: waitlist, psychoeducation/supportive intervention, standard care/treatment as usual, pharmacotherapy, placebo, and other psychotherapeutic intervention (i.e., active treatment–when this was the case, we coded for the specific psychotherapeutic intervention, i.e., CT, behavioral intervention like exposure, relaxation).
- Participants’ age category as identified by the authors: children, adolescents, and adults.
- Participants’ clinical status: nonclinical/asymptomatic, subclinical/symptomatic‐undiagnosed, clinical/diagnosed, remitted/treated‐asymptomatic.
- Treatment delivery format number: individual versus group intervention.
- Number of treatment hours: continuous moderator.

### 1.3 Meta‐analytic procedure

#### 1.3.1 Effect size computation

For each comparison between REBT condition and every condition group, we computed the effect size (ES) indicating the difference between the two groups at postintervention or used the ES reported in the original study (when the study reported ESs). To compute ESs, we used means and standard deviations whenever they were available; when this was not the case, we used available statistics, such as *t* and *F* values and sample sizes, *p* values, and degrees of freedom. Separate ESs were computed for postintervention and follow‐up measurements. For every study, we computed the mean ES, by averaging all the individual ESs for each outcome reported for that study at a certain assessment point (i.e., postintervention, follow‐up). All the ESs were coded such as positive values of Cohen's *d* indicated a better condition for the participants in the REBT intervention group compared with a specific control group. Analyses were computed using the Comprehensive Meta‐Analysis software (version 2.2.046).

We used the study sample as the unit of the analysis or subgroups of the sample (e.g., males and females) for articles where data were reported as such. However, for certain moderation analyses that implied including different data coming from the same study in different categories (e.g., the case of studies reporting comparisons between REBT and multiple control groups, or studies including different types of outcome measures), we assumed independence of comparison or outcomes for those analyses.

We computed two major sets of ESs: ESs for between‐group comparisons (i.e., measures comparing REBT intervention with a control group) and ESs for within‐group comparisons (i.e., measures comparing pre‐ and post‐REBT intervention variables). We chose to do this because we wanted to cover the field of REBT interventions as extensively as possible, using the data from clinical trials and pre‐post designs as well. In this sense, previous meta‐analyses also considered within‐group data (e.g., Johnsen & Friborg, 2015; Lyons & Woods, 1991). Within each type of comparison (between and within groups), we calculated separate sets of ESs for outcome measures (i.e., measures of symptoms or other outcome variables) and alleged mechanisms of change measures (i.e., rational and irrational beliefs, theoretically assumed to mediate the change in outcome variables), both for postintervention and follow‐up assessments. Separated analyses were conducted for each of these types of ESs coming from between‐group and within‐group comparisons.

We computed the mean ES using a random effects model, which assumes that studies come from a pool where the ESs vary in the population (Riley, Higgins, & Deeks, 2011). Homogeneity of ESs was assessed with the *Q* statistic (which compares true heterogeneity to random error, with statistically significant values indicating true heterogeneity beyond random error) and the *I^2^* index (which reflects the percentage of observed heterogeneity; Borenstein, Hedges, Higgins, & Rothstein, 2009). Values of *I^2^* around 0% indicate no heterogeneity, values of 25% indicate low levels of heterogeneity, and values around 50% indicate moderate levels, while values of 75% and above indicate high heterogeneity (Higgins, Thompson, Deeks, & Altman, 2003).

To assess publication bias, for each data set based on which we generated a pooled ES, we first created and visually inspected funnel plots, which graphically contrasts standard error for each study (determined by sample size) against the study's ES (Light, Pillemer, & Wilkinson, 1984). Next, we applied Duval and Tweedie's (2000) trim‐and‐fill procedure to each data set. This procedure estimates the likely number of missing studies on the basis of asymmetry in the funnel plot, yielding corrected ESs and confidence intervals adjusted to account for these missing studies (Borenstein et al., 2009; Duval & Tweedie, 2000).

Categorical moderators were tested with a mixed‐effects meta‐analytic test, which pools the studies within a category using a random effects model, while testing for significant differences between groups using a fixed effect model. For each moderator, a category was included in the analysis only when *k* (the number of studies / samples in that category) was at least 2 because calculating ESs for less than that would lead to meaningless results. However, we reported ESs for outcome categories, regardless on the number of studies employing those outcomes. For testing continuous moderators, we used unrestricted maximum likelihood meta‐regression analysis. Where multiple continuous predictors were introduced in the analysis, we used ordinary least square regression (OLS) analysis weighted by sample size, instead of meta‐regression.

### 1.4 Quality assessment

The quality of the included studies was assessed using the eight criteria from Cuijpers, van Straten, Bohlmeijer, Hollon, and Andersson (2010). These criteria are based on the framework of empirically supported psychotherapies proposed by Chambless and Hollon (1998) and follow the recommendations proposed by the Cochrane Collaboration to assess the methodological validity of a study (Higgins & Green, 2006). A study was considered of high quality if (a) participants were diagnosed using a standardized clinical interview, (b) a treatment manual was used, (c) the therapists who treated patients were adequately trained, (d) treatment integrity had been checked, (e) data were analysed using intent‐to‐treat procedure, (f) the study had sufficient statistical power to detect at least a large effect size, (g) adequate randomization had been employed, and (h) assessors were blinded to the participant's condition. Each of the eight criteria was judged as being met/unmet or was marked as unclear (when insufficient information was provided). We also coded separately whether the studies were randomized or nonrandomized (i.e., as reported by the authors) and used this distinction as a moderator (two independent evaluators had to agree 100% for each criterion).

## 2 RESULTS

### 2.1 Study characteristics

**Figure 1:**
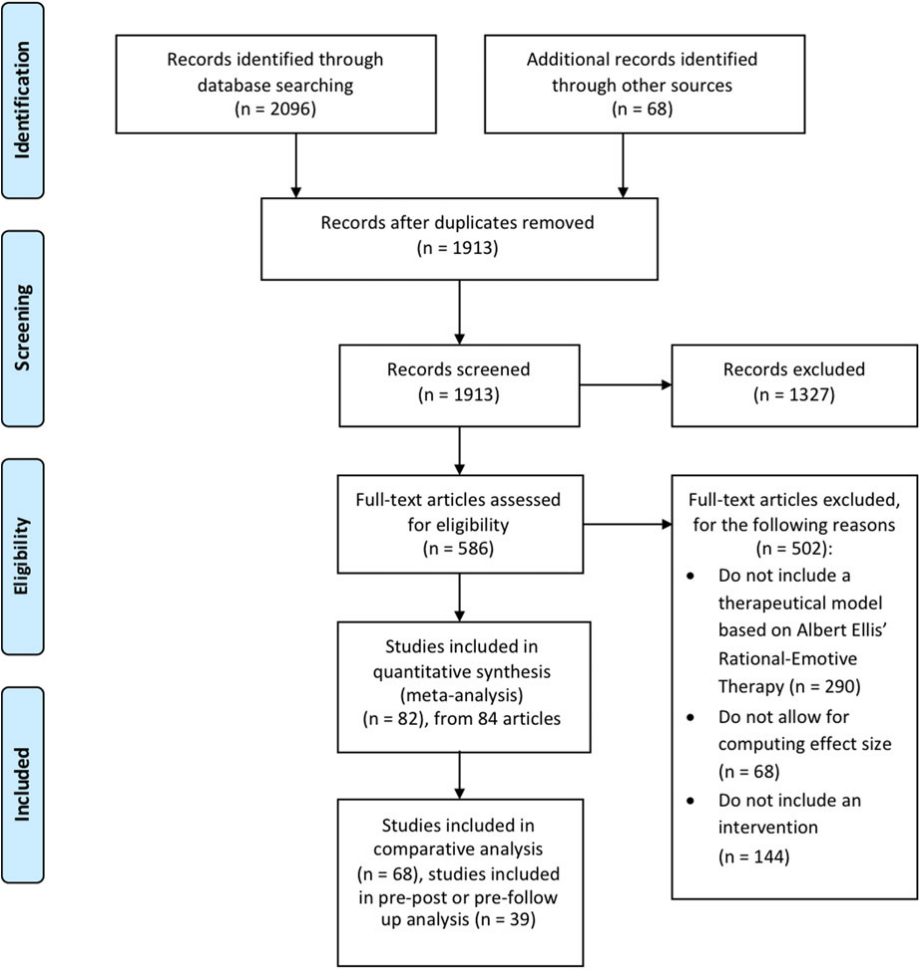
PRISMA flow chart for manuscript identification, selection, and inclusion

The PRISMA Flow Chart (Moher, Liberati, Tetzlaff, & Altman, The PRISMA Group, 2009) of the entire selection process is shown in Figure 1.

Characteristics of the studies included in the meta‐analysis are summarized in Table S1 available upon request from the authors. All 68 studies (*k =* 77 samples) comparing REBT with a control group reported outcome measures, while only 34 studies (*k* = 38 samples) reported measures of alleged mechanisms of change. Out of the studies reporting outcome measures, 55 (*k* = 62) were psychotherapy studies, 9 (*k* = 10) were REBT educational interventions, and 4 (*k* = 5) were counseling studies. Out of the 68 studies, 21 (*k* = 25) were nonrandomized and 47 (*k* = 52) reported random assignment of participants across conditions.

### 2.2 Between‐group analyses

#### 2.2.1 Outcome effect sizes

##### Overall ESs

The overall effect size of REBT interventions (all between‐group comparisons combined; *k* = 77), computed from posttest measurements, was *d* = .58, 95% confidence interval [CI] [.44, .71]. Heterogeneity was high and significant, *Q*(76) = 246.38, *p* < .001, *I^2^* = 69.15. Effect sizes for different types of outcomes are shown in Table 1. Trim‐and‐fill analysis (Duval & Tweedie, 2000) indicated no missing studies to the left of the mean, which would have reduced the ES. Higher ESs were in favor of randomized trials (*k* = 52), *Q*_between_ (1) = 5.67, *p* = .017, these obtaining medium effects, *d* = .69, 95% CI [.50, .88], while nonrandomized trials (*k* = 25) obtained an overall small ES, *d* = .39, 95% CI [.23, .55].

**Table 1:** Between groups analysis–overall effect sizes for different types of outcomes at posttest

Outcome category	*k*	*d*	95% CI	*Q* within	*I^2^*
Emotional outcomes	55	0.52***	[0.35, 0.68]	171.67***	68.55
Anger	6	0.59**	[0.24, 0.94]	2.54	0.00
Anxiety	38	0.45***	[0.23, 0.66]	113.94***	67.53
Depression	19	0.54**	[0.20, 0.88]	95.09***	81.07
Distress	16	0.94***	[0.54, 1.33]	116.44***	87.12
Behavioral outcomes	13	0.56***	[0.25, 0.87]	43.32***	72.30
Cognitive outcomes	29	0.32**	[0.11, 0.54]	85.89***	67.40
Health outcomes	8	0.57**	[0.22, 0.93]	12.67	44.75
Other outcomes	21	0.47***	[0.26, 0.69]	45.90***	56.42
Psychophysiological outcomes	8	0.52	[−0.05, 1.09]	31.30***	77.64
Quality of life	8	0.69*	[0.14, 1.25]	27.84***	74.86
School performance	8	0.86*	[0.18, 1.54]	48.15***	85.46
Social skills	6	0.04	[−0.27, 0.36]	3.39	0.00

*Note*. CI = confidence interval.

*p < .05. **p < .01. ***p < .001.

**Table 2:** Within group analysis–overall effect sizes for different types of outcomes at posttest

Outcome	*k*	*d*	95% CI	*Q* within	*I^2^*
Emotional outcomes	29	0.56***	[0.41, 0.70]	92.24***	69.64
Anger	2	0.88**	[0.33, 1.42]	1.29	22.60
Anxiety	19	0.63***	[0.46, 0.81]	40.62**	55.68
Depression	10	0.57***	[0.29, 0.86]	28.11**	67.98
Distress	7	0.34**	[0.11, 0.56]	19.16**	68.69
Behavioral outcomes	9	0.33***	[0.18, 0.49]	16.44*	51.35
Cognitive outcomes	13	0.57***	[0.33, 0.81]	37.71***	68.18
Health outcomes	5	0.79***	[0.49, 1.09]	1.63	0.00
Other outcomes	9	0.52**	[0.19, 0.86]	40.34***	80.17
Psychophysiological outcomes	4	0.32	[−0.08, 0.71]	6.2	51.62
Quality of life	7	0.52**	[0.19, 0.86]	17.13**	64.98
School performance	1	0.72*	[0.06, 1.39]	–	–
Social skills	5	0.40*	[0.04, 0.76]	8.68	53.91

*Note*. CI = confidence interval.

*p <.05. **p < .01. ***p < .001.

The overall ESs for REBT interventions at follow‐up (*k* = 29) was *d* = 0.66, 95% CI [.37, .95]. Heterogeneity was high and significant, *Q*(28) = 130.10, *p* < .001, *I^2^* = 78.48. ESs for different types of outcomes at follow‐up are reported in Table S2 available upon request from the authors. Trim and fill suggested no missing studies due to publication bias. Again, ES was higher for randomized trials (*k* = 23), *d* = .74, 95% CI [.38, 1.10], than for nonrandomized studies (*k* = 6), *d* = .38, 95% CI [.10, .66], but the difference is not statistically significant.

The high heterogeneity in the overall ESs might be expected given that the overall ES and the analyses for specific outcomes combine data from studies implementing different types of REBT interventions (i.e., psychotherapy, counseling, and educational interventions), different type of data report, different number of sessions, etc. Thus, in the next analyses we specifically looked at several moderators in order reduce heterogeneity and allow readers to better understand how effective REBT in more specific contexts defined by these variables is.

#### 2.2.2 Moderation analyses

We found no moderation effect for type of REBT intervention and type of data report, neither at postintervention nor at follow‐up. Also, no significant moderation was evident when we compared ESs for different age categories, participants’ clinical status, and intervention delivery format. A meta‐regression analysis (*k* = 71) showed that the number of sessions positively predicted outcome ESs at posttest, *B* = .02, *Z* = 2.18, *p* = .026, but not at follow‐up.

**Figure 2:**
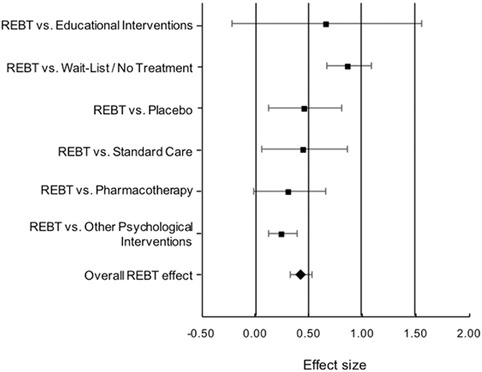
Forest plot of posttest ESs stratified on comparison/control group

Type of the control group significantly moderated ESs for both postintervention, *Q*(5) = 25.21, *p* < .001, and follow‐up measurements *Q*(4) = 11.77, *p* = .019. More specifically, at postintervention, we obtained the following ESs: REBT versus waitlist/no treatment (*k* = 41): *d* = .88, 95% CI [.67, 1.01]; REBT versus placebo (*k* = 13): *d* = .47, 95% CI [.12, .81]; REBT versus treatment as usual/standard care (*k* = 7): *d* = .46, 95% CI [.06, .86]; REBT versus psychoeducation/supportive intervention (*k* = 7): *d* = .67, 95% CI [−.22, 1.55]; REBT versus pharmacotherapy (*k* = 5): *d* = .32, 95% CI [−.02, .66], and REBT versus other psychological interventions (*k* = 36): *d* = .26, 95% CI [.13, .39]. These results are displayed in a forest plot (see Figure 2). At follow‐up, ESs were positive and significant when comparing REBT with waitlist/no treatment (*k* = 13), *d* = 1.16, 95% CI [.55, 1.77], psychoeducation/supportive intervention (*k* = 5), *d* = 1.25, 95% CI [.14, 2.36], and other psychological interventions (*k* = 17), *d* = .46, 95% CI [.13, .79]. There was just one study comparing REBT with pharmacotherapy at follow‐up, favoring REBT, and thus was not included in the analysis. The comparisons with standard care (*k* = 2) and placebo (k = 5) yielded positive (favoring REBT) but not significant ESs; however, the number of studies is too small to draw rigorous conclusions for these analyses).

We also attempted to see whether the quality and year of publication predicted ESs using weighted ordinary least squares (OLS) regression analysis. For outcomes at postintervention, we found that the quality of the studies negatively predicted ESs, *β* = −.06, *p* = .001, while year of publication was a significant positive predictor, *β* = .07, *p* < .001. When both predictors where entered in the same model of the outcome ESs, they both kept their signs and were significant, *β* = .11, *p* < .001, for year of publication and *β* = −.10, *p* < .001, for quality of the studies. At follow‐up, study quality alone negatively predicted the outcome ESs, *β* = −.20, *p* < .001, while year of publication was not significant. When both predictors were entered in the model, both quality, *β* = −.36, *p* < .001, and year, *β* = .25, *p* < .001, were significant predictors, but of opposite signs.

#### 2.2.3 Complementary analyses

As data showed, REBT being more effective than other psychological interventions, but equally effective as psychoeducation/supportive intervention, we suspected that participants’ clinical status may be a confounding variable (as psychoeducation interventions are more likely to involve subclinical and nonclinical participants, while psychotherapy is more likely to involve clinical participants). Consequently, we re‐ran this moderation analysis considering only studies that involved clinical participants and reported outcome measurements postintervention (*k* = 29). We found a significant moderation effect, *Q*(4) = 19.84, *p* = .001. REBT yielded higher ESs compared to waitlist/no treatment (*k* = 6), *d* = 1.44, 95% CI [.52, 2.37], placebo condition (*k* = 2), *d* = .83, 95% CI [.55, 1.10], and treatment as usual/standard care (*k* = 4), *d* = .73, 95% CI [.11, 1.36]. However, ESs were nonsignificant (still favoring REBT) when REBT was compared to pharmacotherapy (*k* = 4), *d* = .32, 95% CI [−.10, .74] or other psychological interventions (*k* = 13), *d* = .15, 95% CI [−.07, .38]. There were no studies comparing REBT to psychoeducation in clinical populations.

We also computed the ESs for the comparisons between REBT and other CBT interventions. Compared to cognitive interventions (including CT/CBT and stress inoculation) the effect size at post‐test (k = 9) was d = .08, 95% CI [−.10; .25] and at follow‐up (k = 6) d = .05, 95% CI [−.14; .24]. Compared to behavioral interventions (including skills training, exposure, and desensitization) (k = 15) d = .07, 95% CI [−.12; .26] at post test and (k = 8): d = .19, 95% CI [−.08; .46] at follow‐up.

#### 2.2.4 ESs for alleged mechanisms of change

##### Overall ESs

For posttest measurements (*k* = 38), results indicated an overall ES of REBT interventions on both irrational and rational beliefs of *d* = .70, 95% CI [.43, .98], *Q*(37) = 253.18, *p* < .001, *I^2^* = 85.39. The trim‐and‐fill analysis indicated no publication bias, with the adjusted values identical to the initial one. For follow‐up measurements (*k* = 15), results indicated an overall ES of *d* = .57, 95% CI [.16, .98], *Q*(14) = 67.12, *p* < .001, *I^2^* = 79.14. The trim‐and‐fill analysis indicated no publication bias. The ES for randomized studies was d = .89, 95% CI [.45, .1.34], while for non‐randomized studies was d = .52 [.20, .85], but the difference between these categories was not significant. At follow‐up the ES decreased to medium and small respectively, and the difference between randomized and non‐randomized studies was still not significant.

An OLS regression analysis, using ESs for alleged mechanisms of change as the dependent variable, showed that the quality of the studies did not predict ESs at posttest, but the year of publication did, *β* = .09, *p* < .001. When put together in the same model, year remained a positive predictor, *β* = .09, *p* < .001, while quality of the studies was still not significant. At follow‐up, quality was a strong and negative predictor *β* = −.63, *p* < .001. Year remained a positive predictor of rational and irrational beliefs ESs at follow−up, *β* = .19, *p* < .001 for year. Taken together in the same model their predictive power increased, quality of the studies remained a negative predictor, *β* = −.82, *p* < .001, and *β* = .50, *p* < .001 and year of publication remained a positive predictor.

#### 2.2.5 Moderation analyses

For alleged mechanisms of change, we ran moderation analyses only at posttest (*k* = 38) because there were few studies measuring rational or irrational beliefs at follow‐up (*k* = 15) and the number of studies in each cell for most of the analyses would have been too small to generate meaningful results. At posttest, we obtained a significant moderation effect for type of REBT intervention, *Q*(2) = 7.93, *p* = .019, which emerged from a large and significant effect for educational interventions *k* = 6, *d* = .82, 95 % CI [.42, 1.21], a medium effect for psychotherapeutic interventions, *k* = 30, *d* = .74, 95 % CI [.41, 1.07], and a negative, but not significant effect for counseling interventions, *k* = 2, *d* = −.31, 95 % CI [−1.02, .40]. Participants’ age category, clinical status, or treatment delivery format did not moderate the ESs for alleged mechanisms.

Type of data report could not be tested as a moderator because all measures were self‐report. Type of the control group significantly moderated the ES, *Q*(5) = 11.87, *p* = 0.037, with REBT interventions yielding higher ESs when compared to waitlist/no treatment, *k* = 18, *d* = 1.35, 95 % CI [.82, 1.88], treatment as usual/standard care, *k* = 3, *d* = .33, 95% CI [.02, .65], other psychological intervention, *k* = 19, *d* = .37, 95 % CI [.02, .72], and psychoeducation/supportive intervention, *k* = 4, *d* = .48, 95% CI [.17, .80], but nonsignificant when compared to placebo, *k* = 3, *d* = .58, 95 % = [−.41, 1.57] (still favoring REBT). There were only two studies comparing REBT to pharmacotherapy and the difference was not significant*, d* = .32, 95 % CI [−.25, .90] (favoring REBT). Number of treatment sessions did not predict the effect of REBT on rational and irrational beliefs.

#### 2.2.6 Association between alleged mechanisms of change and outcomes ESs

To investigate if rational and irrational beliefs relate to outcomes (as mechanisms of change should), we computed several meta‐regressions using ESs on the proposed mechanisms as predictor and the ESs of the outcomes as dependent variables. We computed such an analysis for both between and within comparisons as well as both time points–posttest and follow‐up. Results for between comparisons indicated a significant association between ESs on mechanisms and overall outcomes, *B* = .38, *Z* = 6.75, *p* < .001 at postintervention (two extreme values were removed for this analysis after examining the plot), and at follow‐up, *B* = .43, *Z* = 2.66, *p* = .008.

### 2.3 Within‐group analyses

#### 2.3.1 Outcome ESs

##### Overall ESs

From pre‐ to post‐REBT interventions (*k* = 40), results showed an overall ES of *d* = .56, 95% CI [.43, .69], with significant heterogeneity, *Q*(39) *=* 133.74, *I^2^* = 70.84. Duval and Tweedie's (2000) trim‐and‐fill procedure showed 13 missing studies to the left of the mean, which would have reduced the ES to *d* = .39, 95% CI [.26, .51]. ESs for different outcome categories at posttest form the within‐subject analysis are shown in Table 2.

For follow‐up measurements (*k* = 18), we obtained an overall within‐group ES of *d* = .46, 95% CI [.34, .57], with no significant heterogeneity, *Q*(17) *=* 13.36, *p* = .712, *I^2^* = .00. Duval and Tweedie's (2000) trim‐and‐fill procedure estimated eight trimmed studies, adjusting the ES to 0.37, 95% CI [.26, .49]. ESs for different outcome categories at follow‐up are available on request, in Table S3.

#### 2.3.2 Moderation analyses

Type of intervention moderated the effect of REBT from pre‐ to posttest, *Q*(1) = 36.25, *p* < .001, with psychotherapy studies (*k* = 37) showing a medium ES of *d* = .65, 95% CI [.51, .79], while self‐help interventions (*k* = 2) showing a small ES, but still significant, *d* = .12, 95% CI [.01, .22]. All other categories within type of intervention had just one study and were not included in this analysis. Looking at type of data report at postintervention (self‐report, *k* = 37; other‐report, *k* = 8; objective, *k* = 11; clinician‐rated, k = 2) we found a moderation effect, *Q*(3.00) = 9.70, *p* = .021, with studies using self‐report and objective measures obtaining medium ESs, *d* = .59, 95% CI [.46, .73], and *d* = .48, 95% CI [.30, .65], respectively, studies using other‐report measures obtaining small ESs, *d* = .29, 95% CI [.14, .44], and studies usign clinician‐rated measures obtaining a large but not significant ES, d = 2.33 95% CI [−2.04; 6.70].

Participants’ age category moderated postintervention ESs, *Q*(2) = 6.35, *p* = .042. Studies conducted on adults (*k* = 33) yielded a significant medium ES, *d* = .59, 95% CI [.44, .73], as well as those conducted on adolescents (*k* = 2), *d* = .46, 95% CI [0.19, 0.73], while those conducted on children (*k* = 3) yielded a nonsignificant ES, *d* = .20, 95% CI [−.08, .47]. There was no moderation effect for clinical status of participants and treatment delivery format.

For follow‐up measurement, we could not analyze the effect of type of intervention because of the lack of studies in other categories than psychotherapeutic interventions. We did not found a significant effect for any of the moderators we tested: participant's age category, clinical status of participants, treatment delivery format, and type of data report.

#### 2.3.3 ESs for alleged mechanisms of change

##### Overall ESs

From pre‐ to postintervention (*k* = 20), we found an overall ES of *d* = .61, *p* < .001, 95% CI [.36, .85]. Heterogeneity was high and significant, *Q*(19) = 97.11, *p* < .001, *I^2^* = 80.44. Duval and Tweedie's (2000) trim‐and‐fill analysis identified five missing studies, which would have reduced the ES to *d* = .42, 95% CI [.20, .72]. For follow‐up measurements (*k* = 3), the overall ES was *d* = .33, *p* < .001, 95% CI [.11, .56], with no evidence of heterogeneity, *Q*(2) = 1.15, *p* = 0.563, *I^2^* = 0.00. Trim‐and‐fill analysis estimated two studies missing, rendering the ES to *d* = .24, 95% CI [.05, .43].

#### 2.3.4 Moderation analyses

We conducted moderation analyses only on postintervention data (*k* = 20) because few studies collected follow‐up measurements (*k* = 3). Type of REBT intervention could not be used as a moderator because, except for studies involving psychotherapy (*k* = 18), there was only one study in each of the other categories. Type of data report was also not tested as a moderator here because all the measurements were self‐report. Neither participants’ age category nor clinical status of participants moderated postintervention ESs. However, treatment delivery format significantly moderated the mean ES postintervention, *Q*(2) = 8.29, *p* = .016, with group interventions (*k* = 14) yielding a significant medium ES, *d* = .51, 95% CI [.23, .78], while mixed interventions (*k* = 3) indicated a high ES *d* = 1.11, 95% CI [.70, 1.52]. Individual interventions yielded a small and nonsignificant ES (*k* = 3), *d* = .32, 95% CI [−.07, .77].

#### 2.3.5 Alleged mechanisms of change and outcomes

Results for within comparisons indicated a significant association between ESs on mechanisms of change and overall outcomes at postintervention, *B* = .42, *Z* = 3.39, *p* < .001, and a nonsignificant association at follow‐up, *B* = .74, *Z* = .80, *p* = .42, but only three studies were included in the follow‐up analysis.

## 3 DISCUSSION

### 3.1 Main effects

Overall, our results showed, in most of the cases, medium and significant ESs of REBT, both between and within groups, for outcomes and mechanisms of change at postintervention and follow‐up. The sole exceptions were the ESs for outcomes and mechanisms in the within‐subjects analysis at follow‐up, which were still significant, but small in magnitude. It is worth noting that these analyses were based on the smallest number of studies. Some non‐significant ESs were found for analyses were the number of subjects was low, but these effects were still favoring REBT. The heterogeneity in the data was very high in most cases. Although this is not unexpected, given the extended period of time we covered, wide diversity of REBT interventions considered (delivered in different formats), wide diversity of control groups, and wide range of outcomes, results need to be carefully interpreted. More specifically, we further presented the results in separate outcome categories, to allow for clinically valid interpretations of results (as an overall ES representing divergent outcomes and comparisons would not be sufficiently informative).

When comparing REBT intervention with a control group, (more relevant for the efficacy logic) and looking at the ESs for different types of outcomes, REBT generated high ESs (minimum ES of *d* = .80) for distress and school performance at posttest, and behavioral outcomes, health outcomes, and school performance at follow‐up. REBT generated medium ES (minimum ES of *d* = .50) for anger, behavioral outcomes, depression, emotional outcomes, health outcomes, and quality of life at posttest, and for distress, depression, and overall emotional outcomes at follow‐up. Finally, REBT generated small but significant ESs (minimum ES of *d* = .20, 95% CI not including 0) for anxiety, cognitive outcomes, and other types of outcomes at posttest, and quality of life and other types outcomes at follow‐up. However, we acknowledge that some outcome categories (such as anger or social skills) included a limited number of studies, and this does not allow for strong conclusions to be derived.

When comparing pre‐ to posttest REBT intervention measures, within groups, (potentially more relevant for the effectiveness logic) we found an overall medium ES postintervention in symptoms. More specifically, we found high ESs for REBT (minimum ES of *d* = .80) for anger pre‐post and psychophysiological outcomes pre‐follow‐up (but we only had two, respectively one study in this category). We found medium ESs (minimum ES of *d* = .50) for anxiety, cognitive outcomes, depression, emotional outcomes, health outcomes, quality of life, school performance, and other outcomes pre‐post, and for anxiety, distress, health outcomes, and quality of life pre‐follow‐up. Finally, we found small ESs (minimum ES of *d* = .20, 95% CI not including 0) for behavioral outcomes, distress, and social skills pre‐post, and for cognitive outcomes and depression from pre‐intervention to follow‐up.

Previous meta‐analyses (Lyons & Woods, 1991) reported a much larger overall ES of REBT interventions compared to baseline scores (i.e., *d* = 1.37). However, they included fewer studies (including unpublished reports) and used multiple ESs per study (i.e. assuming independence of the different outcomes within a study) when computing the mean ES, which might have artificially inflated their results.

In terms of alleged mechanisms of change, our results showed medium ESs, both in cases when REBT was compared to a control group and within REBT groups, with the exception of within‐subjects follow‐up ES, which was small but significant. However, less than half of the studies reported measures of rational and/or irrational beliefs at postintervention, in between group analyses, and less than half of those which report did so at follow‐up. The same was true for studies that compared pre‐ to postintervention scores, with the mention that only three of those studies reported follow‐up measurements on alleged mechanisms of change. As expected, REBT yielded higher ESs on alleged mechanisms of change when compared to waitlist, treatment as usual/standard care, other psychological interventions, and psychoeducation, given the fact that it specifically targets irrational and rational beliefs. However, differences in ES were nonsignificant when comparing REBT to placebo and pharmacotherapy, probably to the small number of studies involving these comparisons (three and two, respectively).

Also, while the ES for change in irrational beliefs compared to waitlist/no treatment was large (ES *d* = 1.35), it was quite low (although statistically significant) when compared to treatment as usual and other psychological interventions. This may seem surprising because it appears that irrational beliefs change in a similar manner (i.e., to REBT) in response to other psychological interventions. Nevertheless, previous data, both experimental (e.g., Szentagotai et al., 2008) and meta‐analytic (e.g., Cristea et al., 2015), have shown that dysfunctional thinking changes similarly in CBT compared to other interventions, their modification being less specific than predicted by theory. This points to the idea that dysfunctional thinking (and/or irrational beliefs) can be changed by different approaches, whether they restructure dysfunctional thinking (and/or irrational beliefs) directly or not.

ESs on mechanisms of change were significantly associated with outcome ESs, both between (at postintervention and at follow‐up) and within groups (at postintervention). This means higher ESs in terms of mechanisms of change translate into higher ESs on outcomes, thus potentially pointing to a causal link between the two. Number of sessions predicted outcome ESs in a positive direction, meaning that longer interventions associate with higher ESs. However, this relation was no longer significant for mechanisms of change, probably also because we had a smaller number of studies reporting them.

Although randomized trials obtained higher ESs when compared to nonrandomized studies overall, the quality of the studies negatively predicted ESs at postintervention and at follow‐up–a similar finding reported in other meta‐analyses of CBT as well (e.g. Cuijpers et al., 2013). This finding may seem contradictory at first sight; however, it may be explained by the fact that, although crucial, randomization is only one aspect of the quality of a study. Importantly, randomization may control for the preexistent differences between groups, ensuring groups equivalence. By randomization, we avoid situations in which more affected groups are treated and compared with less affected groups–or vice versa–situations that would artificially decrease or inflate the obtained ESs. But a high‐quality study requires more than this (i.e., a clear protocol, flawlessly implemented, blinding the participants and the assessors to the conditions), including independent randomization, which was not reflected in our simple split between randomized and nonrandomized trials. When quality criteria are simultaneously considered, the effect of the nonspecific therapeutic factors are minimized, leading to smaller but arguably more informative ESs, attributable to the actual, specific effects of the tested intervention.

Moreover, we only coded a quality criterion as positive if that specific information was presented (e.g. if the study did not report how randomization was conducted, we did not code this criterion positively), which may have led to an artificially smaller number of higher quality studies reported. Additionally, some of the quality criteria may possess disputable definitions or imply arbitrary cutoffs (see Cuijpers et al., 2010).

Interestingly, year of publication was a positive predictor, at both postintervention and follow‐up, even when considering quality in the same regression model (which was a negative predictor). So, it seems that more recent studies yield higher ESs, possibly because of the fact that recent studies are more focused on clinical populations, where ESs are expected to be higher. Also, they might have used more effective procedures (documented by previous research), which could have increased the ESs. Although some meta‐analytic data have found publication year to be a negative predictor of ES in CBT studies for depression (Johnsen & Friborg, 2015), other accounts have found year a less consistent predictor relation of outcomes in between‐group comparisons (Cristea et al., 2017).

### 3.2 Moderator effects

ESs were not moderated by type of intervention (i.e., psychotherapy, educational interventions, counseling). This could be explained by the fact that different forms of interventions targeted different participant groups (e.g., psychotherapy usually addresses clinical problems, counseling nonclinical participants) and are consequently associated with specific outcome measures. On the other hand, REBT is rather homogenous in scope and techniques, aiming to change the same categories of irrational beliefs regardless of clinical status or disorder; thus, it is possible that the interventions, regardless of their form, are quite similar. Also, it seems that specific REBT interventions targeted for specific groups are equally efficient for those groups, as we did not find a moderation effect of the clinical status of the participants.

ESs computed based on the post‐REBT intervention outcome measures, between‐groups comparisons, was moderated only by the type of control group, with REBT yielding larger and significant ESs when compared to waitlist, placebo, treatment as usual/standard care, or other psychological interventions, but nonsignificant ESs when compared to psychoeducation or pharmacotherapy. In most cases, “other psychological interventions” referred to CT or specific behavioral interventions (e.g., exposure, relaxation). Because the lack of ES significance when REBT was compared to psychoeducation seemed suspicious (taking into considerations that REBT appeared to be superior to other psychological interventions), we verified if the clinical status of participants acted as a confounded variable here. Because there were no studies comparing REBT with psychoeducation in clinical samples, we speculate that the equivalence between REBT and psychoeducation only stands for nonclinical samples. This is presumable because in nonclinical populations, the space of improvement in terms of intervention outcomes is minimal. In other words, baseline measurements of the outcomes in the nonclinical population are already close to optimal. Therefore, the specific effect of REBT might not have had the chance to appear. The “space of improvement” hypothesis is supported by the fact that, even if significant, the obtained ESs for REBT versus different types of control in subclinical/nonclinical participants were only medium, compared with clinical participants, where we obtained large ESs.

When only clinical participants were considered, results showed higher ESs for REBT versus waitlist/no treatment, placebo, and treatment as usual/standard care comparisons, but nonsignificant ESs when REBT was compared to pharmacotherapy or other psychological interventions. These findings are congruent to those reported by previous meta‐analyses focusing specifically on REBT (e.g., Engels et al., 1993; Lyons & Woods, 1991), and are close to results obtained for CBT, in general, in treating clinical patients (e.g., Cuijpers et al., 2013). Our results suggest that when used with clinical participants, REBT is equally effective as other psychological interventions or pharmacotherapy. This is an important finding because it confirms the clinical utility of REBT.

## 4 CONCLUSION

Overall, the current meta‐analysis indicates that REBT interventions (psychotherapy, educational, or counseling interventions) are efficacious/ effective for various conditions, regardless of clinical status, age of sample, and delivery format, though, as expected, ESs are moderated by type of control condition. Also, REBT interventions are efficacious/ effective when analyzing their effect on alleged mechanisms of change. Because studies are highly heterogeneous in scope, outcomes, and quality of reporting, more studies need to be conducted in the more recent and more rigorous efficacy paradigm, involving various diagnostic categories (and transdiagnostic interventions as well). Last but not least, we need more psychometrically sound instruments to uniformly measure REBT mechanisms of change, and more studies employing mechanisms of change analyses to further test the REBT change theory.

## Supporting information

Table S1 Studies Included in the Meta‐Analysis, Coding Criteria and Effect Sizes for Outcomes Overall (not including mechanisms of change ^a^
Table S2 Between Groups Analysis ‐ Overall Effect Sizes for Different Types of Outcomes at Follow‐Up
Table S3 Within Group Analysis ‐ Overall Effect Sizes for Different Types of Outcomes at Follow‐Up

## References

[jclp22514-bib-0001] Borenstein, M. , Hedges, L. V. , Higgins, J. , & Rothstein, H. R. (2009). Introduction to meta‐analysis. Chichester: John Wiley & Sons.

[jclp22514-bib-0002] Chambless, D. L. , & Hollon, S. D. (1998). Defining empirically supported therapies. Journal of Consulting and Clinical Psychology, 66(1), 7–18. 9489259 10.1037//0022-006x.66.1.7

[jclp22514-bib-0003] Cristea, I. A. , Huibers, M. J. , David, D. , Hollon, S. D. , Andersson, G. , & Cuijpers, P. (2015). The effects of cognitive behavior therapy for adult depression on dysfunctional thinking: A meta‐analysis. Clinical Psychology Review, 42, 62–71. 26319193 10.1016/j.cpr.2015.08.003

[jclp22514-bib-0004] Cristea, I. A. , Stefan, S. , Karyotaki, E. , David, D. , Hollon, S. D. , & Cuijpers, P. (2017). The effects of cognitive behavioral therapy are not systematically falling: A revision of Johnsen and Friborg (2015). Psychological Bulletin, 143(3), 326–340. 28230413 10.1037/bul0000062

[jclp22514-bib-0005] Cuijpers, P. , Berking, M. , Andersson, G. , Quigley, L. , Kleiboer, A. , & Dobson, K. S. (2013). A meta‐analysis of cognitive‐behavioural therapy for adult depression, alone and in comparison with other treatments. Canadian Journal of Psychiatry, 58(7), 376–385. 23870719 10.1177/070674371305800702

[jclp22514-bib-0006] Cuijpers, P. , van Straten, A. , Bohlmeijer, E. , Hollon, S. D. , & Andersson, G. (2010). The effects of psychotherapy for adult depression are overestimated: A meta‐analysis of study quality and effect size. Psychological Medicine, 40(2), 211–223. 19490745 10.1017/S0033291709006114

[jclp22514-bib-0007] David, D. (2014). Rational emotive behavior therapy. New York: Oxford University Press.

[jclp22514-bib-0008] David, D. , Lynn, S. J. , & Ellis, A. (2010). Rational and irrational beliefs: Research, theory, and clinical practice. Oxford: Oxford University Press.

[jclp22514-bib-0009] David, D. , & Montgomery, G. H. (2011). The scientific status of psychotherapies: A new evaluative framework for evidence‐based psychosocial interventions. Clinical Psychology: Science and Practice, 18(2), 89–99.

[jclp22514-bib-0010] David, D. , Szentagotai, A. , Lupu, V. , & Cosman, D. (2008). Rational emotive behavior therapy, cognitive therapy, and medication in the treatment of major depressive disorder: a randomized clinical trial, posttreatment outcomes, and six‐month follow‐up. Journal of Clinical Psychology, 64(6), 728–746. 18473339 10.1002/jclp.20487

[jclp22514-bib-0011] DiGiuseppe, R. A. , Doyle, K. A. , Dryden, W. , & Backx, W. (2013). A practitioner's guide to rational‐emotive behavior therapy. Oxford: Oxford University Press.

[jclp22514-bib-0012] Duval, S. , & Tweedie, R. (2000). Trim and fill: a simple funnel‐plot–based method of testing and adjusting for publication bias in meta‐analysis. Biometrics, 56(2), 455–463. 10877304 10.1111/j.0006-341x.2000.00455.x

[jclp22514-bib-0013] Ellis, A. (1995). Changing rational‐emotive therapy (RET) to rational emotive behavior therapy (REBT). Journal of Rational‐Emotive & Cognitive‐Behavior Therapy, 13(2), 85–89.

[jclp22514-bib-0014] Emmelkamp, P. M. , & Beens, H. (1991). Cognitive therapy with obsessive‐compulsive disorder: A comparative evaluation. Behaviour Research and Therapy, 29(3), 293–300. 1679324 10.1016/0005-7967(91)90120-r

[jclp22514-bib-0015] Engels, G. I. , Garnefski, N. , & Diekstra, R. F. (1993). Efficacy of rational‐emotive therapy: A quantitative analysis. Journal of Consulting and Clinical Psychology, 61(6), 1083–1090. 8113487 10.1037//0022-006x.61.6.1083

[jclp22514-bib-0016] Gaviţa, O. A. , David, D. , Bujoreanu, S. , Tiba, A. , & Ionuţiu, D. R. (2012). The efficacy of a short cognitive–behavioral parent program in the treatment of externalizing behavior disorders in Romanian foster care children: Building parental emotion‐regulation through unconditional self‐and child‐acceptance strategies. Children and Youth Services Review, 34(7), 1290–1297.

[jclp22514-bib-0017] Gonzalez, J. E. , Nelson, J. R. , Gutkin, T. B. , Saunders, A. , Galloway, A. , & Shwery, C. S. (2004). Rational emotive therapy with children and adolescents a meta‐analysis. Journal of Emotional and Behavioral Disorders, 12(4), 222–235.

[jclp22514-bib-0018] Higgins, J. P. , Thompson, S. G. , Deeks, J. J. , & Altman, D. G. (2003). Measuring inconsistency in meta‐analyses. BMJ, 327(7414), 557–560. 12958120 10.1136/bmj.327.7414.557 PMC192859

[jclp22514-bib-0019] Higgins, J. P. T. , & Green, S. (2006). The Cochrane Library. 4. Chichester, UK: John Wiley & Sons, Ltd.

[jclp22514-bib-0020] Johnsen, T. J. , & Friborg, O. (2015). The effects of cognitive behavioral therapy as an anti‐depressive treatment is falling: A meta‐analysis. Psychological Bulletin, 141(4), 747–768. 25961373 10.1037/bul0000015

[jclp22514-bib-0021] Joyce, M. R. (1995). Emotional relief for parents: Is rational‐emotive parent education effective? Journal of Rational‐Emotive and Cognitive‐Behavior Therapy, 13(1), 55–75.

[jclp22514-bib-0022] Light, R. J. , Pillemer, D. B. , & Wilkinson, I. (1984). Summing up: The science of reviewing research. Cambridge: Harvard University Press.

[jclp22514-bib-0023] Lyons, L. C. , & Woods, P. J. (1991). The efficacy of rational‐emotive therapy: A quantitative review of the outcome research. Clinical Psychology Review, 11(4), 357–369.

[jclp22514-bib-0024] Meaden, A. , Keen, N. , Aston, R. , Barton, K. , & Bucci, S. (2013). Cognitive therapy for command hallucinations: An advanced practical companion. New York: Routledge.

[jclp22514-bib-0025] Mersch, P. P. A. , Emmelkamp, P. M. , Bögels, S. M. , & Van der Sleen, J. (1989). Social phobia: Individual response patterns and the effects of behavioral and cognitive interventions. Behaviour Research and Therapy, 27(4), 421–434. 2775152 10.1016/0005-7967(89)90013-2

[jclp22514-bib-0026] Moher, D. , Liberati, A. , Tetzlaff, J. , & Altman, D. G. (2009). Preferred reporting items for systematic reviews and meta‐analyses: The PRISMA statement. Annals of Internal Medicine, 151(4), 264–269. 19622511 10.7326/0003-4819-151-4-200908180-00135

[jclp22514-bib-0027] Montgomery, G. H. , David, D. , Kangas, M. , Green, S. , Sucala, M. , Bovbjerg, D. H. , … Schnur, J. B. (2014). Randomized controlled trial of a cognitive‐behavioral therapy plus hypnosis intervention to control fatigue in patients undergoing radiotherapy for breast cancer. Journal of Clinical Oncology, 32(6), 557–563. 24419112 10.1200/JCO.2013.49.3437 PMC3918539

[jclp22514-bib-0028] Riley, R. D. , Higgins, J. P. , & Deeks, J. J. (2011). Interpretation of random effects meta‐analyses. British Medical Journal, 342, d549. 21310794 10.1136/bmj.d549

[jclp22514-bib-0029] Sava, F. A. , Yates, B. T. , Lupu, V. , Szentagotai, A. , & David, D. (2009). Cost‐effectiveness and cost‐utility of cognitive therapy, rational emotive behavioral therapy, and fluoxetine (prozac) in treating depression: A randomized clinical trial. Journal of Clinical Psychology, 65(1), 36–52. 19051275 10.1002/jclp.20550

[jclp22514-bib-0030] Schnur, J. B. , David, D. , Kangas, M. , Green, S. , Bovbjerg, D. H. , & Montgomery, G. H. (2009). A randomized trial of a cognitive‐behavioral therapy and hypnosis intervention on positive and negative affect during breast cancer radiotherapy. Journal of Clinical Psychology, 65(4), 443–455. 19226611 10.1002/jclp.20559 PMC2756503

[jclp22514-bib-0031] Shapiro, D. A. , & Shapiro, D. (1982). Meta‐analysis of comparative therapy outcome studies: A replication and refinement. Psychological Bulletin, 92(3), 581–604. 7156259

[jclp22514-bib-0032] Szentagotai, A. , David, D. , Lupu, V. , & Cosman, D. (2008). Rational emotive behavior therapy versus cognitive therapy versus pharmacotherapy in the treatment of major depressive disorder: Mechanisms of change analysis. Psychotherapy: Theory, Research, Practice, Training, 45(4), 523. 10.1037/a0014332 22122538

[jclp22514-bib-0033] Thorpe, G. L. , Freedman, E. G. , & McGalliard, D. W. (1984). Components of rational‐emotive imagery: Two experiments with nonassertive students. Journal of Rational Emotive Therapy, 2(2), 11–19.

[jclp22514-bib-0034] Trip, S. , Vernon, A. , & McMahon, J. (2007). Effectiveness of rational‐emotive education: a quantitative meta‐analytical study. Journal of Cognitive and Behavioral Psychotherapies, 7(1), 81–93.

