# Supplementary material for: 50 years of rational‐emotive and cognitive‐behavioral therapy: A systematic review and meta‐analysis
Source: J Clin Psychol. 2017 Sep 12;74(3):304–18. doi: 10.1002/jclp.22514 (PMC5836900; doi:10.1002/jclp.22514)
Supplement: Supplementary file 1 — Table S1 Studies Included in the Meta‐Analysis, Coding Criteria and Effect Sizes for Outcomes Overall (not including mechanisms of change a Table S2 Between Groups Analysis ‐ Overall Effect Sizes for Different Types of Outcomes at Follow‐Up Table S3 Within Group Analysis ‐ Overall Effect Sizes for Different Types of Outcomes at Follow‐Up [file JCLP-74-304-s001.docx]

**50 YEARS OF REBT**

**Supplementary Materials**

Table S1

*Studies Included in the Meta-Analysis, Coding Criteria and Effect Sizes for Outcomes Overall (not including mechanisms of change) ^a^*

| **Article** | **Type of sample** | **Outcomes** | **Specific type of intervention** | **Specific type of comparison** | **Type of measurement** | **Follow up interval** | ***d* at post-test between** | ***d* at follow-up between** |
| --- | --- | --- | --- | --- | --- | --- | --- | --- |
|  |  |  |  |  |  |  | ***d* at post-test within** | ***d* at follow-up within** |
| Aghataher & Mahani, 2014 | subclinical | Depression; Cognitive outcomes | REBT + pharmacotherapy | Pharmacotherapy | Self-report | none | 0.48 | - |
|  |  |  |  |  |  |  | - | - |
| Baither & Godsey, 1979 | non-clinical | Anxiety | REBT | Other Psychotherapy, Relaxation; Wait-list / No treatment | Self-report | none | 0.26 | - |
|  |  |  |  |  |  |  | 0.18 | - |
| Barabasz & Barabasz, 1981 subgroup 1 | non-clinical | Psychophysiological; Anxiety | RET | Placebo; Wait-list / No treatment | Objective; Self-report | none | 1.29** | - |
|  |  |  |  |  |  |  | - | - |
| Barabasz & Barabasz, 1981 subgroup 2 | non-clinical | Psychophysiological; Anxiety | RET | Placebo; Wait-list / No treatment | Objective; Self-report | none | 1.15* | - |
|  |  |  |  |  |  |  | - | - |
| Block, 1978 | non-clinical | Behavioral outcomes; School performance | REE | Educational intervention, Psychodynamic education - Human relationships training; Other Psychotherapy, Human Relations (psychodynamic); Wait-list / No treatment | Objective; Other report | 4 months | 3.85** | 4.14** |
|  |  |  |  |  |  |  | - | - |
| Block, 1980 | non-clinical | Health outcomes | RET; cognitive restructuring | Other Psychotherapy, Deep muscle relaxation; Wait-list / No treatment | Objective | 1.32 | 1.32* | 3.54** |
|  |  |  |  |  |  |  | - | - |
| Carmody, 1978 | non-clinical | Anxiety; Cognitive outcomes; Social skills | Rational-Emotive Assertion Training | Other Psychotherapy, Behavioral Assertion Training; Other Psychotherapy, Self-Instructional Assertion Training; Wait-list / No treatment | Self-report; Objective | 3 months | 0.17 | 0.06 |
|  |  |  |  |  |  |  | 0.87* |  |
| Cristea, Benga, & Opre, 2008 | non-clinical | Irrational Beliefs; Anxiety | Rational-Emotive and Behavioral Education | Wait-list / No treatment; Placebo | Self-report | none | -0.28 | - |
|  |  |  |  |  |  |  | - | - |
| Cowan & Brunero, 1997 | clinical, anxiety disorders | Anxiety; Depression | REBT | - | Self-report | none | - | - |
|  |  |  |  |  |  |  | 0.97** | - |
| David, Szentagotai, Lupu, & Cosman, 2008;  Szentagotai, David, Lupu, & Cosman, 2008;  Sava, Yates, Lupu, Szentagotai, & David, 2009 | clinical, MDD | Cognitive outcomes; Depression; Irrational Beliefs; Quality of life | REBT | Other Psychotherapy, CT; Pharmacotherapy | Self-report; clinician-rated; Objective | 6 months | 0.08 | 0.19 |
|  |  |  |  |  |  |  | 0.23 | 0.24 |
| David & Matu, 2013 | non-clinical | Distress; Irrational Beliefs; Rational Beliefs; Other | Rational Emotive Coaching | - | Objective; self-report | none | - | - |
|  |  |  |  |  |  |  | 0.25 | - |
| David, 2014 subgroup 1 | subclinical; non-clinical | Behavioral outcomes; Depression; Distress; Rational Beliefs; Irrational Beliefs; Other | Rational parenting program | Standard care; Wait-list / No treatment | Other report; Self-report | 1 month | 0.31 | 0.39 |
|  |  |  |  |  |  |  | 0.27 | 0.28 |
| David, 2014 subgroup2 | subclinical; non-clinical | Behavioral outcomes; Depression; Distress; Rational Beliefs; Irrational Beliefs; Other | Rational parenting program | Standard care; Wait-list / No treatment | Other report; Self-report | 1 month | 0.29 | 0.22 |
|  |  |  |  |  |  |  | - | - |
| Decker & Russell, 1981 | non-clinical | Anxiety; Irrational Beliefs; School performance; Other | Cue-controlled relaxation + cognitive restructuring | Other Psychotherapy, Study Skills Counselling; Wait-list / No treatment | Self-report; Other report | 10 weeks | -0.18 | 0.35 |
|  |  |  |  |  |  |  | - | - |
| DiGiuseppe & Kassinove, 1976 subgroup 1 | non-clinical | Anxiety; Irrational Beliefs; Rational Beliefs; Other | REE | Other psychological intervention; Wait-list / No treatment | Self-report | none | 0.78* | - |
|  |  |  |  |  |  |  | - | - |
| DiGiuseppe & Kassinove, 1976 subgroup 2 | non-clinical | Anxiety; Irrational Beliefs; Rational Beliefs; Other | REE | Other psychological intervention; Wait-list / No treatment | Self-report | none | 0.23 | - |
|  |  |  |  |  |  |  | - | - |
| DiGiuseppe, McGowan, Sutton, & Gardner, 1990 | subclinical | Anger; Anxiety; Behavioral outcomes; Cognitive outcomes; Depression; | REBT | Wait-list / No treatment | Self-report; Objective | none | 0.96^Τ^ | - |
|  |  |  |  |  |  |  | 0.64 ^Τ^ | - |
| Drazen, Nevid, Pace, & O’Brien, 1982 | subclinical | Psychophysiological | RET/AT (assertiveness training) | Other Psychotherapy, Anxiety management training; Other Psychotherapy, Hypertension education counseling | Objective | 8 weeks | 0.58 | 0.44 |
|  |  |  |  |  |  |  | 1.06^Τ^ | 0.87^Τ^ |
| Emmelkamp & Beens, 1991 | clinical, OCD | Anxiety; Depression; Irrational Beliefs | RET + exposure | Other Psychotherapy, Exposure | Clinician-rated; Other report; Self-report | 6 months | 0.20 | 0.28 |
|  |  |  |  |  |  |  | - | - |
| Emmelkamp, Mersch, Vissia, & van der Helm, 1985 | clinical, social phobia | Anxiety; Irrational Beliefs; Health outcomes ; Psychophysiological | RET | Other Psychotherapy, Exposure; Other Psychotherapy, Self-instructional training | Self-report; Objective | 1 month | 0.32 | - |
|  |  |  |  |  |  |  | 0.51 | 0.58 |
| Everaerd & Dekker, 1985 subgroup a | clinical, male sexual dysfunction | Health outcomes; Quality of life | RET | Other Psychotherapy, Sex therapy | Self-report | 6 months – 1 year | 0.62 | 0.77 |
|  |  |  |  |  |  |  | 1.19^Τ^ | 1.16 |
| Everaerd & Dekker, 1985 subgroup b | clinical, male sexual dysfunction | Health outcomes; Quality of life | RET | Other Psychotherapy, Sex therapy | Self-report | 6 months – 1 year | 0.57 | 0.86 |
|  |  |  |  |  |  |  | 0.78 | 0.64 |
| Fadaei et al., 2011 | non-clinical | Other | REBT | Wait-list / No treatment | Self-report | none | 1.44** | - |
|  |  |  |  |  |  |  | 2.19** | - |
| Fava, Rafanelli, Cazzaro, Conti, & Grandi, 1998 | remitted patients, mixed diagnoses: depression, anxiety disorders | Anger; Anxiety; Depression; Health outcomes; Quality of life; Other | Well-being therapy | Other Psychotherapy, CBT | Self-report; Clinician-rated | none | 0.33 | - |
|  |  |  |  |  |  |  | 0.87 ^Τ^ | - |
| Flanagan, Povall, Dellino, & Byrne, 1998 | non-clinical | Irrational Beliefs; Social skills | REBT + Problem Solving | Other Psychotherapy, Problem-solving | Self-report; Other report | none | 0.19 | - |
|  |  |  |  |  |  |  | -0.14 | - |
| Fuller, Digiuseppe, O’Leary, Fountain, & Lang, 2010 | clinical, Axis I & Axis II disorders | Anger; Depression | REBT | - | Self-report | none | - | - |
|  |  |  |  |  |  |  | 1.24** | - |
| Galanter, Egelko, & Edwards, 1993 subgroup 1 | clinical, alcohol dependence | Behavioral outcomes; Distress; Rational Beliefs | Smart/rational recovery | Wait-list / No treatment | Self-report | none | 0.48** | - |
|  |  |  |  |  |  |  | 0.10 | - |
| Galanter, Egelko, & Edwards, 1993 subgroup 2 | clinical, alcohol dependence | Behavioral outcomes; Distress; Rational Beliefs | Smart/rational recovery | Wait-list / No treatment | Self-report | none | 0.48** | - |
|  |  |  |  |  |  |  | 0.15 | - |
| Gavita, David, Bujoreanu, Tiba, & Ionutiu, 2012 | subclinical | Behavioral outcomes; Distress; Other | Short Enhanced Cognitive–Behavioral Parent Training | Wait-list / No treatment | Other report | none | 0.77** | - |
|  |  |  |  |  |  |  | - | - |
| Haynes et al., 1983 a | non-clinical | Anxiety; School performance | Rational Emotive Counselling | Other Psychotherapy, Self-instruction training; Placebo | Self-report; Other report | none | 0.26 | - |
|  |  |  |  |  |  |  | - | - |
| Haynes et al., 1983 b | non-clinical | Anxiety; School performance | Rational Emotive Counselling | Other Psychotherapy, Self-instruction training; Placebo | Self-report; Other report | none | 1.04 | - |
|  |  |  |  |  |  |  | - | - |
| Holroyd, 1976 | subclinical | Anxiety; Behavioral outcomes; Distress; School performance | RET; RET + exposure | Other Psychotherapy, Exposure; Placebo; Wait-list / No treatment | Self-report; Objective; Other report | 1 month | 0.52 | 0.53 |
|  |  |  |  |  |  |  | 0.47 | 0.77^Τ^ |
| Horan, 1996 | non-clinical | Cognitive outcomes; Irrational Beliefs | RET | Other Psychotherapy, Relaxation | Self-report | none | 0.12 | - |
|  |  |  |  |  |  |  | - | - |
| Hymen & Warren, 1978 | subclinical | Anxiety; Behavioral outcomes; Cognitive outcomes; Irrational Beliefs; School performance | RET | - | Objective;   other report ; self-report | 1 month | ^-^ | - |
|  |  |  |  |  |  |  | 0.85^Τ^ | 0.42 |
| Iftene, Predescu, Stefan, & David, 2015 | clinical, MDD | Cognitive outcomes; Depression; Distress; Psychophysiological | REBT + pharmacotherapy; REBT | Pharmacotherapy | Self-report; Objective | none | 0.03 | - |
|  |  |  |  |  |  |  | - | - |
| Jalali, Maussavi, Yazdi, & Fadardi, 2014 | clinical, late blindness | Anxiety; Cognitive outcomes; Depression; Distress; Irrational Beliefs; Psychophysiological | REBT | Wait-list / No treatment | Self-report | 1 month | 3.98** | 3.86** |
|  |  |  |  |  |  |  | - | - |
| Jaycox, Reivich, Gillham, & Seligman, 1994 | subclinical | Behavioral outcomes; Cognitive outcomes; Depression; Distress | Penn Prevention Programme | Wait-list / No treatment | Other report; Self-report | 6 months | 0.20 | 0.26 |
|  |  |  |  |  |  |  | 0.36^Τ^ | 0. 34^Τ^ |
| Johnson, & Ridley, 1992 | subclinical | Cognitive outcomes; Depression; Irrational Beliefs | RET | - | Self-report | none | - | - |
|  |  |  |  |  |  |  | 1.58 ^Τ^ | - |
| Kachman & Mazer, 1990 | non-clinical | Behavioral outcomes; Cognitive outcomes; Rational Beliefs; School performance; Other | REE added to psychology classes | Educational intervention, Regular psychology classes | Other report; Objective; Self-report | none | 0.26 | - |
|  |  |  |  |  |  |  | - | - |
| Kanter & Goldfried, 1979 | non-clinical | Anxiety; Cognitive outcomes; Irrational Beliefs; Psychophysiological | Rational Restructuring; Rational Restructuring + self-control desensitization | Other Psychotherapy, Exposure; Wait-list / No treatment | Self-report; Objective | 9 weeks | 0.56 | 0.40 |
|  |  |  |  |  |  |  | 0.69* | - |
| Kassinove, Miller, & Kalin, 1980 | clinical , neurotic | Anxiety; Rational Beliefs; Other | REBT | Wait-list / No treatment | Self-report | none | 0.39 | - |
|  |  |  |  |  |  |  | - | - |
| Keller, Croake, & Brooking, 1975 | non-clinical | Anxiety; Irrational Beliefs | RET | - | Self-report | none | - | - |
|  |  |  |  |  |  |  | 0.97* | - |
| Kim, Kim, & Kim, 2015 | non-clinical | Cognitive outcomes; Other | REBT | Wait-list / No treatment | Self-report | None | 0.56 | - |
|  |  |  |  |  |  |  | - | - |
| Kirkby, 1994 | subclinical | Anxiety; Depression; Distress; Irrational Beliefs | Coping skills training | Placebo; Wait-list / No treatment | Self-report | 9 months | 0.49 | 0.41 |
|  |  |  |  |  |  |  | - | - |
| Kushnir, Malkinson, & Ribak, 1994 | non-clinical | Irrational Beliefs; Other | REE | Placebo | Self-report | none | 0.43 | - |
|  |  |  |  |  |  |  | 0.32 | - |
| LaConte, Shaw, & Dunn, 1993 | non-clinical | Cognitive outcomes; Behavioral outcomes | Rational Emotive Affective Education | Wait-list / No treatment | Other report; Self-report | none | -0.14 | - |
|  |  |  |  |  |  |  | - | - |
| Lent, Russell, & Zamostny, 1981 | subclinical | Anxiety | Systematic Rational Restructuring | Placebo; Wait-list / No treatment | Self-report | 8 weeks | -0.02 | -0.02 |
|  |  |  |  |  |  |  | 0.64^Τ^ | 0. 64^Τ^ |
| Lipsky, Kassinove, & Miller, 1980 | clinical; adjustment reaction of adulthood, neurosis | Anxiety; Distress; Rational beliefs; Other | REBT; REBT + Rational Role Reversal; REBT + Rational Emotive Imagery (guided imagery on rational and irrational beliefs) | Other psychological intervention; Wait-list / No treatment | Self-report | none | 1.57* | - |
|  |  |  |  |  |  |  | - | - |
| Macaskill & Macaskill, 1996 | clinical, MDD | Depression; Irrational Beliefs; Other | RET + pharmacotherapy | Pharmacotherapy | Clinician-rated; Self-report | none | 1.07^Τ^ | - |
|  |  |  |  |  |  |  | - | - |
| Maes & Schloesser, 1988 | clinical, asthma | Anger; Anxiety; Depression; Health outcomes; Other | RET + pharmacotherapy | Pharmacotherapy | Self-report; Objective | none | 0.79 | - |
|  |  |  |  |  |  |  | - | - |
| Mahfar, Aslan, Noah, Ahmad, & Jaafar, 2014 | non-clinical | Distress; Irrational Beliefs | REBT | Wait-list / No treatment | Self-report | none | 1.95** | - |
|  |  |  |  |  |  |  | - | - |
| Malkiewich & Merluzzi, 1980 subgroup a | subclinical | Anxiety; Cognitive outcomes | REBT | Other Psychotherapy, Systematic desensitization; Wait-list / No treatment | Self-report | none | 0.61 | - |
|  |  |  |  |  |  |  | - | - |
| Malkiewich & Merluzzi, 1980 subgroup b | subclinical | Anxiety; Cognitive outcomes | REBT | Other Psychotherapy, Systematic desensitization; Wait-list / No treatment | Self-report | none | 0.76 | - |
|  |  |  |  |  |  |  | - | - |
| Malouff, Lanyon, & Schutte, 1988 | subclinical | Depression; Distress | REBT | Other Psychotherapy, problem solving | Self-report | 1 month | 0.80^Τ^ | 0.09 |
|  |  |  |  |  |  |  | - | - |
| Mattick & Peters, 1988 | clinical, social anxiety | Anxiety; Cognitive outcomes; Distress; Irrational Beliefs | REBT | Other Psychotherapy, Exposure | Other report; Self-report | 3 months | -0.28 | 0.12 |
|  |  |  |  |  |  |  | - | - |
| Maxwell & Wilkerson, 1982 | non-clinical | Anxiety; Other | RT | - | Self-report | - | - | - |
|  |  |  |  |  |  |  | 0.55^Τ^ | - |
| McNaughton-Cassill, Bostwick, Arthur, Robinson, & Neal, 2002 subgroup 1 | non-clinical | Anxiety; Cognitive outcomes; Depression; Irrational Beliefs; Other | REBT | Standard care | Self-report | none | 0.39 | - |
|  |  |  |  |  |  |  | 0.14 | - |
| McNaughton-Cassill, Bostwick, Arthur, Robinson, & Neal, 2002 subgroup 2 | clinical, infertility | Anxiety; Cognitive outcomes; Depression; Irrational Beliefs; Other | REBT | Standard care | Self-report | none | -0.10 | - |
|  |  |  |  |  |  |  | 0.25 | - |
| Mersch, 1995 | clinical, social anxiety | Anxiety; Cognitive outcomes; Irrational Beliefs | REBT (social skills included) | Other Psychotherapy, Exposure | Self-report | 3 months | -0.06 | 0.02 |
|  |  |  |  |  |  |  | - | - |
| Mersch, Emmelkamp, Bogels, & van der Sleen, 1989 subgroup a | clinical, social phobia | Anxiety; Behavioral outcomes; Cognitive outcomes; Quality of life; Rational Beliefs; Social skills | RET | Other Psychotherapy, Social skills training | Self-report; Objective; Other report | none | -0.19 | - |
|  |  |  |  |  |  |  | 0.44 | - |
| Mersch, Emmelkamp, Bogels, & van der Sleen, 1989 subgroup b | clinical, social phobia | Anxiety; Behavioral outcomes; Cognitive outcomes; Quality of life; Rational Beliefs; Social skills | RET | Other Psychotherapy, Social skills training | Self-report; Objective; Other report | none | 0.09 | - |
|  |  |  |  |  |  |  | 0.57^Τ^ | - |
| Miller & Kassinove, 1978 | non-clinical | Anxiety; Distress; Irrational Beliefs | REE | Wait-list / No treatment | Self-report | none | 0.97* | - |
|  |  |  |  |  |  |  | - | - |
| Möller & Botha, 1996 | non-clinical | Irrational Beliefs; Other | REBT | Wait-list / No treatment | Self-report; Other report | 10 weeks | 0.66 | - |
|  |  |  |  |  |  |  | 0.33 | 0.30 |
| Montgomery et al., 2009 | clinical, cancer radiotherapy patients | Health outcomes | REBT + hypnosis | Other Psychotherapy, Standard care | Self-report | none | 0.69^Τ^ | - |
|  |  |  |  |  |  |  | - | - |
| Montgomery et al., 2014 | clinical, cancer radiotherapy patients | Health outcomes | REBT + hypnosis | Placebo | Self-report | 4-week follow up; 6-months follow up | 0.87** | 0.77** |
|  |  |  |  |  |  |  | - | *- |
| Munjack et al., 1984 | subclinical | Quality of life | RET | Wait-list / No treatment | Self-report | 6-9 months | 4.50** | - |
|  |  |  |  |  |  |  | 0.71 | 0.55^Τ^ |
| Navarro et al., 1992 | clinical, drug abuse | Quality of life | RET | - | Self-report | none | - | - |
|  |  |  |  |  |  |  | 0.78** | - |
| Nielsen et al., 1996 | subclinical | Irrational Beliefs; Other | RET | - | Self-report; Other report | none | - | - |
|  |  |  |  |  |  |  | 0.41* | - |
| Nolan, Mattis, & Holliday, 1970 | subclinical | Distress | Systematic Desensitization + Rational component | - | Self-report | 12 months | - | - |
|  |  |  |  |  |  |  | 0.72^Τ^ | 1.05^Τ^ |
| Reardon & Tosi, 1977 | clinical, deliquency | Depression; Cognitive outcomes | Rational Stage Directed Imagery | Placebo; Wait-list / No treatment | Self-report | 2 months | 0.53 | 0.64 |
|  |  |  |  |  |  |  | 0.84^Τ^ | 0.84^Τ^ |
| Rosenbhaum, McMurray, & Campbell, 1991 subgroup 1 | non-clinical | Rational Beliefs; Cognitive outcomes | REE | Educational intervention, Attentional control | Self-report | none | 0.28 | 0.29 |
|  |  |  |  |  |  |  | - | - |
| Rosenbhaum, McMurray, & Campbell, 1991 subgroup 2 | non-clinical | Rational Beliefs; Cognitive outcomes | REE | Educational intervention, Attentional control | Self-report | none | 0.83^Τ^ | 0.91^Τ^ |
|  |  |  |  |  |  |  | - | - |
| Schnur, et al., 2009 | clinical, breast cancer | Distress | REBT + hypnosis | standard care | Self-report | none | 1.00* | - |
|  |  |  |  |  |  |  | - | - |
| Scholing & Emmelkamp, 1993 | clinical, social phobia | Anxiety; Cognitive outcomes; Depression | REBT | Other Psychotherapy, Exposure | Self-report | 3 months | 0.07 | 0.16 |
|  |  |  |  |  |  |  | 0.39 | 0.39 |
| Scholing & Emmelkamp, 1996 | clinical, social anxiety | Anxiety; Cognitive outcomes; Health outcomes | REBT | - | Self-report | 18 months | - | - |
|  |  |  |  |  |  |  | 1.03** | 0.67* |
| Shannon & Allen, 1998 | non-clinical | School performance | Perception Analysis Training | Placebo | Other report | none | 0.78* | - |
|  |  |  |  |  |  |  | - | - |
| Shelley, Battaglia, Lucey, Ellis, & Opler, 2001 | clinical, schizophrenia | Distress | REBT | standard care | Self-report | none | 1.37** | - |
|  |  |  |  |  |  |  | - | - |
| Stanton, 1989 | non-clinical | Distress ; Rational Beliefs | RET | Other psychological intervention | Self-report | 12 months | 0.94* | 1.10** |
|  |  |  |  |  |  |  | 1.02** | 0.87** |
| Tafrate & Kassionove, 1998 | subclinical | Anger; Psychophysiological; Other | Rational self-statements + exposure | Other Psychotherapy, irrational self-statements; Other Psychotherapy, irrelevant self-statements | Objective; Other report; Self-report | none | 0.69 | - |
|  |  |  |  |  |  |  | - | - |
| Thorpe, Freedman, & McGalliard, 1984 study 1 | subclinical | Anxiety; Cognitive outcomes; Irrational Beliefs; Social skills | Cognitive rehearsal of rational statements; Behavioral rehearsal of target response | Other Psychotherapy, Behavioral rehearsal of target response; Other Psychotherapy, Emotional rehearsal of upsetting events | Self-report; Other report | none | 0.23 | - |
|  |  |  |  |  |  |  | - | - |
| Thorpe, Freedman, & McGalliard, 1984 study 2 | subclinical | Anxiety; Cognitive outcomes; Irrational Beliefs; Social skills | Behavioral rehearsal + cognitive restructuring | Other Psychotherapy, Behavioral rehearsal; Other Psychotherapy, Behavioral rehearsal + emotional rehearsal; Other Psychotherapy, Cognitive restructuring + emotional rehearsal | Self-report; Other report | none | -0.15 | - |
|  |  |  |  |  |  |  | - | - |
| Thurman, 1985a | subclinical | Anger; Irrational Beliefs; Rational Beliefs; Other | Cognitive-behavior modification | Educational intervention, Minimal psychoeducation | Other report; Self-report | none | 0.62 | - |
|  |  |  |  |  |  |  | - | - |
| Thurman, 1985b | subclinical | Anger; Irrational Beliefs; Other | Cognitive-behavior modification | Educational intervention, Minimal psychoeducation | Self-report | 6 months; 1 year | - | 0.74 |
|  |  |  |  |  |  |  | - | - |
| Tiegerman & Kassinove, 1977 | non-clinical | Anxiety ; Other | REBT | Other psychological intervention; Placebo; Wait-list / No treatment | Self-report | none | 0.20 | - |
|  |  |  |  |  |  |  | - | - |
| Todirita & Lupu, 2013 | non-clinical | Other | REE | Educational intervention, Information on gambling, biases that gamblers have; Wait-list / No treatment | Objective | none | -0.21 | - |
|  |  |  |  |  |  |  | - | - |
| Trexler & Karst, 1972 | subclinical | Anxiety; Behavioral outcomes; Irrational Beliefs; Psychophysiological | RET | Other Psychotherapy, Relaxation; Wait-list / No treatment | Self-report; Objective | 2 weeks; 6 weeks; 8 weeks | 0.45 | 0.66 |
|  |  |  |  |  |  |  | 0.79** | 0.52* |
| Warren, McLellarn, & Ponzoha, 1988 | subclinical | Anger; Anxiety; Cognitive outcomes; Depression; Rational Beliefs | RET | Other Psychotherapy, CBT; Wait-list / No treatment | Self-report | 6 months | 0.04 | -0.53 |
|  |  |  |  |  |  |  | - | - |
| Wessel & Mersch, 1994 | subclinical | Anxiety; Cognitive outcomes; Quality of life; Rational Beliefs | RET + in vivo exposure | Wait-list / No treatment | Self-report | none | 0.91* | - |
|  |  |  |  |  |  |  | - | - |
| Wilde, 1994 | subclinical | Cognitive outcomes; Depression; Irrational Beliefs | Let's Get Rational board game | Wait-list / No treatment | Self-report | none | 0.19 | - |
|  |  |  |  |  |  |  | - | - |
| Zelie, Stone, & Lehr, 1980 | non-clinical | Behavioral outcomes; School performance; Other | RBT | Wait-list / No treatment | Other report; Objective | none | 1.28* | - |
|  |  |  |  |  |  |  | - | - |

*Note:* CBT = Cognitive Behavioral Therapy; RBT = Rational Behavior Therapy; REBT = Rational Emotive Behavior Therapy; REE = Rational Emotive Education; RET = Rational Emotive Therapy; RT = Rational Therapy; MDD = Major Depression Disorder.

*Note*: *^a^* There were three studies which only reported effect sizes for mechanisms of change (Breen, Kruedelbach & Walker, 2001; Ray, Freidlander, & Solomon, 1984; Turner, Slater, & Baker, 2014), which we included in the meta-analysis but are not reported here.

*Note*: ^Τ^ *p* < 0.05; * *p* < 0.01; ** *p* < 0.001

Table S2

*Between Groups Analysis* - *Overall Effect Sizes for Different Types of Outcomes at Follow-Up*

| ***Outcome category*** | ***k*** | ***d*** | ***95% CI*** | ***Q within*** | ***I^2^*** |
| --- | --- | --- | --- | --- | --- |
| Emotional outcomes | 20 | 0.51** | [0.17; 0.85] | 83.53*** | 77.25 |
| Anger | 2 | 0.27 | [-0.37; 0.90] | 0.57 | - |
| Anxiety | 13 | 0.47 | [-0.10; 1.04] | 73.96*** | 83.78 |
| Depression | 10 | 0.67* | [0.06; 1.29] | 84.78*** | 89.38 |
| Distress | 8 | 0.75* | [0.01; 1.49] | 74.34*** | 90.58 |
| Behavioral outcomes | 5 | 1.15* | [0.19; 2.12] | 35.41*** | 88.70 |
| Cognitive outcomes | 12 | 0.35 | [-0.11; 0.81] | 60.74*** | 81.89 |
| Health outcomes | 4 | 1.34* | [0.04; 2.63] | 19.23*** | 84.40 |
| Other outcomes | 3 | 0.40* | [0.01; 0.79] | 1.22 | 0.00 |
| Psychophysiological outcomes | 2 | 0.34 | [-0.31; 0.98] | 0.06 | - |
| Quality of life | 3 | 0.43* | [0.05; 0.81] | 1.53 | 0.00 |
| School performance | 1 | 1.89*** | [0.97; 2.81] | - | - |
| Social skills | 1 | 0.24 | [-0.69; 1.16] | - | - |

*Note:* **p* < .05, ***p* < .01, ****p* < .001.

Table S3

*Within Group Analysis* - *Overall Effect Sizes for Different Types of Outcomes at Follow-Up*

| **Outcome** | ***k*** | ***d*** | ***95% CI*** | ***Q within*** | ***I^2^*** |
| --- | --- | --- | --- | --- | --- |
| Emotional outcomes | 11 | 0.47*** | [0.33; 0.61] | 8.11 | 0.00 |
| Anxiety | 6 | 0.52*** | [0.32; 0.73] | 0.55 | 0.00 |
| Depression | 3 | 0.23* | [0.05; 0.41] | 0.66 | 0.00 |
| Distress | 4 | 0.74*** | [0.47; 1.02] | 1.03 | 0.00 |
| Behavioral outcomes | 2 | 0.29 | [-0.01; 0.59] | 0.03 | - |
| Cognitive outcomes | 4 | 0.40* | [0.07; 0.73] | 10.22* | 70.65 |
| Health outcomes | 4 | 0.52** | [0.19; 0.85] | 1.83 | 0.00 |
| Other outcomes | 2 | 0.45 | [-0.03; 0.94] | 1.35 | 25.78 |
| Psychophysiological outcomes | 1 | 0.87* | [0.05; 1.68] | - | - |
| Quality of life | 3 | 0.59* | [0.12; 1.07] | 0.21 | 0.00 |

*Note:* **p* <.05, ***p* < .01, *** *p* < .001.

References Included in the Meta-Analysis

Aghataher, A., & Mahani, K. N. (2014). The effect of Rational Emotive Behavior group Therapy on self-concept and depression of self-introduced drug abusers referred to ofogh addiction treatment center in Zarand (Kerman, Iran). *Biomedical & Pharmacology Journal, 7*(1), 317–323.

Baither, R. C., & Godsey, R. (1979). Rational Emotive Education and relaxation training in large group treatment of test anxiety. *Psychological Reports*, *45*(1), 326–326.

Barabasz, A. F., & Barabasz, M. (1981). Effects of Rational‐Emotive Therapy on psychophysiological and reported measures of test anxiety arousal. *Journal of Clinical Psychology*, *37*(3), 511–514.

Block, J. (1978). Effects of a Rational–Emotive mental health program on poorly achieving, disruptive high school students. *Journal of Counseling Psychology*, *25*(1), 61–65.

Block, J. (1980). Effects of Rational Emotive Therapy on overweight adults. *Psychotherapy: Theory, Research & Practice*, *17*(3), 277–280.

Breen, R. B., Kruedelbach, N. G., & Walker, H. I. (2001). Cognitive changes in pathological gamblers following a 28-day inpatient program. *Psychology of Addictive Behaviors*, *15*(3), 246–248.

Carmody, T. P. (1978). Rational-emotive, self-instructional, and behavioral assertion training: Facilitating maintenance. *Cognitive Therapy and Research, 2,* 241–253.

Cowan, D., & Brunero, S. (1997). Group therapy for anxiety disorders using Rational Emotive Behaviour Therapy. *The Australian and New Zealand Journal of Mental Health Nursing*, *6*(4), 164–168.

Cristea, I. A., Benga, O., & Opre, A. (2008). The Implementation of a Rational-Emotive Educational intervention for anxiety in a 3rd grade classroom: An analysis of relevant procedural and developmental constraints. *Journal of Evidence-Based Psychotherapies*, *8*(1), 31–51.

David, D., Szentagotai, A., Lupu, V., & Cosman, D. (2008). Rational Emotive Behavior Therapy, Cognitive Therapy, and medication in the treatment of major depressive disorder: A randomized clinical trial, posttreatment outcomes, and six-month follow-up. *Journal of Clinical Psychology*, *64*(6), 728–746.

David, O. A. (2014). The rational positive parenting program for child externalizing behavior: Mechanisms of change analysis. *Journal of Evidence-Based Psychotherapies, 14*, 21–38.

David, O. A., & Matu, S. A. (2013). How to tell if managers are good coaches and how to help them improve during adversity? The managerial coaching assessment system and the rational managerial coaching program. *Journal of Cognitive and Behavioral Psychotherapies*, *13*(2a), 259–274.

Decker, T. W., & Russell, R. K. (1981). Comparison of cue-controlled relaxation and cognitive restructuring versus study skills counseling in treatment of test-anxious college underachievers. *Psychological Reports*, *49*(2), 459–469.

DiGiuseppe, R., & Kassinove, H. (1976). Effects of a Rational-Emotive school mental health program on children's emotional adjustment. *Journal of Community Psychology,* *4*, 382–387.

DiGiuseppe, R., McGowan, L., Sutton Simon, K., & Gardner, F. (1990). A comparative outcome study of four cognitive therapies in the treatment of social anxiety. *Journal of Rational-Emotive and Cognitive-Behavior Therapy, 8*, 129–146.

Drazen, M., Nevid, J. S., Pace, N., & O’Brien, R. M. (1982). Worksite-based behavioral treatment of mild hypertension. *Journal of Occupational Medicine: Official Publication of the Industrial Medical Association*, *24*(7), 511–514.

Emmelkamp, P. M., & Beens, H. (1991). Cognitive therapy with obsessive-compulsive disorder: A comparative evaluation. *Behaviour Research and Therapy*, *29*(3), 293–300.

Emmelkamp, P. M., Mersch, P. P., Vissia, E., & van der Helm, M. (1985). Social phobia: A comparative evaluation of cognitive and behavioral interventions. *Behaviour Research and Therapy*, *23*(3), 365–369.

Everaerd, W., & Dekker, J. (1985). Treatment of male sexual dysfunction: Sex therapy compared with systematic desensitization and Rational Emotive Therapy. *Behaviour Research and Therapy*, *23*(1), 13–25.

Fadaei, S., Janighorban, M., Mehrabi, T., Ahmadi, S. A., Mokaryan, F., & Gukizade, A. (2011). Effects of cognitive behavioral counseling on body image following mastectomy. *Journal of Research in Medical Sciences: The Official Journal of Isfahan University of Medical Sciences*, *16*(8), 1047–1054.

Fava, G. A., Rafanelli, C., Cazzaro, M., Conti, S., & Grandi, S. (1998). Well-being therapy: A novel psychotherapeutic approach for residual symptoms of affective disorders. *Psychological Medicine*, *28*(2), 475–480.

Flanagan, R., Povall, L., Dellino, M., & Byrne, L. (1998). A comparison of problem solving with and without Rational Emotive Behavior Therapy to improve children's social skills. *Journal of Rational-Emotive and Cognitive-Behavior Therapy*, *16*(2), 125–134.

Fuller, J. R., Digiuseppe, R., O’Leary, S., Fountain, T., & Lang, C. (2010). An open trial of a comprehensive anger treatment program on an outpatient sample. *Behavioural and Cognitive Psychotherapy*, *38*(4), 485–490.

Galanter, M., Egelko, S., & Edwards, H. (1993). Rational recovery: Alternative to AA for addiction? *The American Journal of Drug and Alcohol Abuse*, *19*(4), 499–510.

Gavita, O. A., David, D., Bujoreanu, S., Tiba, A., & Ionutiu, D. R. (2012). The efﬁcacy of a short cognitive–behavioral parent program in the treatment of externalizing behavior disorders in Romanian foster care children: Building parental emotion-regulation through unconditional self- and child-acceptance strategies. *Children and Youth Services Review 34*, 1290–1297.

Haynes, C. R., Marx, R. W., Martin, J., Wallace, L., Merrick, R., & Einarson, T. (1983). Rational-Emotive counselling and self-instruction training for test anxious high school students. *Canadian Journal of Counselling and Psychotherapy/Revue canadienne de counseling et de psychothérapie*, *18*(1), 31–38.

Holroyd, K. A. (1976). Cognition and desensitization in the group treatment of test anxiety. *Journal of Consulting and Clinical Psychology*, *44*(6), 991–1001.

Horan, J. J. (1996). Effects of computer-based cognitive restructuring on rationally mediated self-esteem. *Journal of Counseling Psychology*, *43*(4), 371–375.

Hymen, S. P., & Warren, R. (1978). An evaluation of Rational-Emotive imagery as a component of Rational-Emotive Therapy in the treatment of test anxiety. *Perceptual and Motor Skills*, *46*(3 Pt 1), 847–853.

Iftene, F., Predescu, E., Stefan, S., & David, D. (2015). Rational-Emotive and Cognitive-Behavior Therapy (REBT/CBT) versus pharmacotherapy versus REBT/CBT plus pharmacotherapy in the treatment of major depressive disorder in youth: A randomized clinical trial. *Psychiatry Research, 225*(3), 687–694.

Jalali, M. D. M., Moussavi, M. S., Yazdi, S. A. A., & Fadardi, J. S. (2014). Effectiveness of Rational Emotive Behavior Therapy on psychological well-being of people with late blindness. *Journal of Rational-Emotive & Cognitive-Behavior Therapy, 32*(4), 233–247.

Jaycox, L. H., Reivich, K. J., Gillham, J., & Seligman, M. E. P. (1994). Prevention of depressive symptoms in school children. *Behavior Research and Therapy, 32*, 801–816.

Johnson, W., & Ridley, C. R. (1992). Brief christian and non‐christian Rational‐Emotive Therapy with depressed christian clients: An exploratory study. *Counseling and Values*, *36*(3), 220–229.

Kachman, D. J., & Mazer, G. E. (1990). Effects of Rational Emotive Education on the rationality, neuroticism and defense mechanisms of adolescents. *Adolescence*, *25*(97), 131–144.

Kanter, N. J., & Goldfried, M. R. (1979). Relative effectiveness of rational restructuring and self-control desensitization in the reduction of interpersonal anxiety. *Behavior Therapy*, *10*(4), 472–490.

Kassinove, H., Miller, N., & Kalin, M. (1980). Effects of pretreatment with Rational Emotive bibliotherapy and Rational Emotive audiotherapy on clients waiting at community mental health center. *Psychological Reports*, *46*, 851–857.

Keller, J. F., Croake, J. W., & Brooking, J. Y. (1975). Effects of a program in rational thinking on anxieties in older persons. *Journal of Counseling Psychology*, *22*(1), 54–57.

Kim, M. A., Kim, J., & Kim, E. J. (2015). Effects of Rational Emotive Behavior Therapy for senior nursing students on coping strategies and self-efficacy. *Nurse Education Today*, *35*(3), 456–460.

Kirkby, R. J. (1994). Changes in premenstrual symptoms and irrational thinking following cognitive-behavioral coping skills training. *Journal of Consulting and Clinical Psychology*, *62*(5), 1026–1032.

Kushnir, T., Malkinson, R., & Ribak, J. (1994). Teaching stress management skills to occupational and environmental health physicians and practitioners. A graduate-level practicum. *Journal of Occupational Medicine: Official Publication of the Industrial Medical Association*, *36*(12), 1335–1340.

Laconte, M. A., Shaw, D., & Dunn, I. D. (1993). The effects of a Rational‐Emotive affective education program for high‐risk middle school students. *Psychology in the Schools*, *30*(3), 274–281.

Lent, R. W., Russell, R. K., & Zamostny, K. P. (1981). Comparison of cue-controlled desensitization, rational restructuring, and a credible placebo in the treatment of speech anxiety. *Journal of Consulting and Clinical Psychology*, *49*(4), 608–610.

Lipsky, M. J., Kassinove, H., & Miller, N. J. (1980). Effects of Rational-Emotive Therapy, rational role reversal, and rational-emotive imagery on the emotional adjustment of community mental health center patients. *Journal of Consulting and Clinical Psychology*, *48*(3), 366–374.

Macaskill, N. D. & Macaskill, A. (1996). Rational-Emotive Therapy plus pharmacotherapy versus pharmacotherapy alone in the treatment of high cognitive dysfunction depression. *Cognitive Therapy and Research, 20*, 575–592.

Maes, S., & Schlösser, M. (1988). Changing health behaviour outcomes in asthmatic patients: A pilot intervention study. *Social Science & Medicine (1982)*, *26*(3), 359–364.

Mahfar, M., Aslan, A. S., Noah, S. M., Ahmad, J., & Jaafar, W. M. W. (2014). Effects of Rational Emotive Education module on irrational beliefs and stress among fully residential school students in Malaysia. *Procedia-Social and Behavioral Sciences*, *114*, 239–243.

Malkiewich, L. E., & Merluzzi, T. V. (1980). Rational restructuring versus desensitization with clients of diverse conceptual levels: A test of a client-treatment matching model. *Journal of Counseling Psychology*, *27*(5), 453–461.

Malouff, J. M., Lanyon, R. I., & Schutte, N. S. (1988). Effectiveness of a brief group RET treatment for divorce-related dysphoria. *Journal of Rational-Emotive and Cognitive-Behavior Therapy*, *6*(3), 162–171.

Mattick, R. P., & Peters, L. (1988). Treatment of severe social phobia: Effects of guided exposure with and without cognitive restructuring. *Journal of Consulting and Clinical Psychology*, *56*(2), 251–260.

Maxwell, J. W., & Wilkerson, J. (1982). Anxiety reduction through group instruction in Rational Therapy. *The Journal of Psychology*, *112*, 135–140.

McNaughton-Cassill, M. E., Bostwick, J. M., Arthur, N. J., Robinson, R. D., & Neal, G. S. (2002). Efficacy of brief couples support groups developed to manage the stress of in vitro fertilization treatment. *Mayo Clinic Proceedings*, *77*(10), 1060–1066.

Mersch, P. P. (1995). The treatment of social phobia: The differential effectiveness of exposure in vivo and an integration of exposure in vivo, Rational Emotive Therapy and social skills training. *Behaviour Research and Therapy*, *33*(3), 259–269.

Mersch, P. P., Emmelkamp, P. M. G., Bogels, S. M., & van der Sleen, J. (1989). Social phobia: Individual response patterns and the effects of behavioral and cognitive interventions. *Behavior Research and Therapy, 27*, 421–434.

Miller, N., & Kassinove, H. (1978). Effects of lecture, rehearsal, written homework, and IQ on the efficacy of a Rational Emotive school mental health program. *Journal of Community Psychology*, *6*(4), 366–373.

Möller, A. T., & Botha, H. C. (1996). Effects of a group Rational-Emotive Behavior Therapy program on the Type A behavior pattern. *Psychological Reports*, *78*(3 Pt 1), 947–961.

Montgomery, G. H., Kangas, M., David, D., Hallquist, M. N., Green, S., Bovbjerg, D., & Schnur, J. B. (2009). Fatigue during breast cancer radiotherapy: An initial randomized study of Cognitive–Behavioral Therapy plus hypnosis. *Health Psychology, 28*, 317–322.

Montgomery, G. H., David, D., Kangas, M., Green, S., Sucala, M., Bovbjerg, D., … Schnur, J. B. (2014). Randomized controlled trial of a Cognitive-Behavioral Therapy plus hypnosis intervention to control fatigue in patients undergoing radiotherapy for breast cancer. *Journal of Clinical Oncology*, *32(6),* 557–563.

Munjack, D. J., Schlaks, A., Sanchez, V. C., Usigli, R., Zulueta, A., & Leonard, M. (1984). Rational-Emotive Therapy in the treatment of erectile failure: An initial study. *Journal of Sex & Marital Therapy*, *10*(3), 170–175.

Navarro, R., Yupanqui, M., Geng, J., Valdivia, G., Girón, M., Rojas, M., … Beletti, A. (1992). Development of a program of behavior modification directed to the rehabilitation of drug-dependent patients: Treatment and follow-up of 223 cases. *The International Journal of the Addictions*, *27*(4), 391–408.

Nielsen, D. M., Horan, J. J., Keen, B., St, P., Carolyn, C., Ceperich, S. D., & Ostlund, D. (1996). An attempt to improve self-esteem by modifying specific irrational beliefs. *Journal of Cognitive Psychotherapy*, *10*(2), 137–149.

Nolan, J. D., Mattis, P. R., & Holliday, W. C. (1970). Long-term effects of behavior therapy: A 12-month follow-up. *Journal of Abnormal Psychology*, *76*(1), 88–92.

Ray, J. B., Freidlander, R. B., & Solomon, G. S. (1984). Changes in rational beliefs among treated alcoholics. *Psychological Reports*, *55*(3), 883–886.

Reardon, J. P., & Tosi, D. J. (1977). The effects of rational stage directed imagery on self-concept and reduction of psychological stress in adolescent delinquent females. *Journal of Clinical Psychology*, *33*(4), 1084–1092.

Rosenbaum, T., McMurray, N. E., & Campbell, I. M. (1991). The effects of rational emotive education on locus of control, rationality and anxiety in primary school children. *Australian Journal of Education*, *35*(2), 187–200.

Sava, F. A., Yates, B. T., Lupu, V., Szentagotai, A., & David, D. (2009). Cost-effectiveness and cost-utility of Cognitive Therapy, Rational Emotive Behavioral Therapy, and fluoxetine (Prozac) in treating depression: A randomized clinical trial. *Journal of Clinical Psychology*, *65*(1), 36–52.

Schnur, J. B., David, D., Kangas, M., Green, S., Bovbjerg, D. H., & Montgomery, G. H. (2009). A randomized trial of a Cognitive-Behavioral Therapy and hypnosis intervention on positive and negative affect during breast cancer radiotherapy*. Journal of Clinical Psychology, 65*, 443–455.

Scholing, A., & Emmelkamp, P. M. (1993). Exposure with and without Cognitive Therapy for generalized social phobia: Effects of individual and group treatment. *Behavior, Research and Therapy, 31*, 667–681.

Scholing, A., & Emmelkamp, P. M. (1996). Treatment of fear of blushing, sweating, or trembling: Results at long-term follow-up. *Behavior Modification*, *20*(3), 338–356.

Shannon, H. D., & Allen, T. W. (1998). The effectiveness of a REBT training program in increasing the performance of high school students in mathematics. *Journal of Rational-Emotive and Cognitive-Behavior Therapy*, *16*(3), 197–209.

Shelley, A. M., Battaglia, J., Lucey, J., Ellis, A., & Opler, L. A. (2001). Symptom-specific group therapy for inpatients with schizophrenia. *Einstein Quarterly Journal of Biology and Medicine, 18*, 21–28.

Stanton, H. E. (1989). Hypnosis and Rational-Emotive Therapy – a de-stressing combination: A brief communication. *The International Journal of Clinical and Experimental Hypnosis*, *37*(2), 95–99.

Szentagotai, A., David, D., Lupu, V., & Cosman, D. (2008). Rational Emotive Behavior Therapy versus Cognitive Therapy versus pharmacotherapy in the treatment of major depressive disorder: Mechanisms of change analysis. *Psychotherapy: Theory, Research, Practice, Training*, *45*(4), 523–538.

Tafrate, R. C., & Kassinove, H. (1998). Anger control in men: Barb exposure with rational, irrational, and irrelevant self-statements. *Journal of Cognitive Psychotherapy*, *12*(3), 187–211.

Thorpe, G. L., Freedman, E. G., & McGalliard, D. W. (1984). Components of Rational-Emotive imagery: Two experiments with nonassertive students. *Journal of Rational Emotive Therapy*, *2*(2), 11–19.

Thurman, C. W. (1985a). Effectiveness of Cognitive-Behavioral treatments in reducing Type A behavior among university faculty. *Journal of Counseling Psychology*, *32*(1), 74–83.

Thurman, C. W. (1985b). Effectiveness of Cognitive–Behavioral treatments in reducing Type A behavior among university faculty: One year later. *Journal of Counseling Psychology*, *32*(3), 445–448.

Tiegerman, S., & Kassinove, H. (1977). Effects of assertive training and cognitive components of Rational Therapy on assertive behaviors and interpersonal anxiety. *Psychological Reports*, *40*(2), 535–542.

Todirita, I. R., & Lupu, V. (2013). Gambling prevention program among children. *Journal of Gambling Studies / Co-Sponsored by the National Council on Problem Gambling and Institute for the Study of Gambling and Commercial Gaming*, *29*(1), 161–169.

Trexler, L. D., & Karst, T. O. (1972). Rational-Emotive therapy, placebo, and no-treatment

Turner, M. J., Slater, M. J., & Barker, J. B. (2014). Not the end of the world: The effects of Rational-Emotive Behavior Therapy (REBT) on irrational beliefs in elite soccer academy athletes. *Journal of Applied Sport Psychology*, *26*(2), 144–156.

Warren, R., McLellarn, R., & Ponzoha, C. (1988). Rational-Emotive Therapy vs general Cognitive-Behavior Therapy in the treatment of low self-esteem and related emotional disturbances. *Cognitive Therapy and Research*, *12*(1), 21–37.

Wessel, I., & Mersch, P. P. A. (1994). A Cognitive-Behavioural group treatment for test-anxious adolescents. *Anxiety, Stress, and Coping*, *7*(2), 149–160.

Wilde, J. (1994). The effects of the “Let's Get Rational” board game on rational thinking, depression, and self-acceptance in adolescents. *Journal of Rational-Emotive and Cognitive-Behavior Therapy*, *12*(3), 189–196.

Zelie, K., Stone, C. I., & Lehr, E. (1980). Cognitive‐Behavioral intervention in school discipline: A preliminary study. *The Personnel and Guidance Journal*, *59*(2), 80–83.
